# Supplementary material for: Accelerated partner therapy (APT) partner notification for people with Chlamydia trachomatis: protocol for the Limiting Undetected Sexually Transmitted infections to RedUce Morbidity (LUSTRUM) APT cross-over cluster randomised controlled trial
Source: BMJ Open. 2020 Mar 29;10(3):e034806. doi: 10.1136/bmjopen-2019-034806 (PMC7170609; doi:10.1136/bmjopen-2019-034806)
Supplement: Supplementary data [file bmjopen-2019-034806supp001.pdf]

[Trust logo]

Your Data Matters to the NHS

Information about your health and care helps us to improve your individual care, speed up diagnosis, plan your local services and research new treatments.

In May 2018, the strict rules about how this data can and cannot be used were strengthened. The NHS is committed to keeping patient information safe and always being clear about how it is used.

You can choose whether your patient information is used for research at [name of clinic].

Version 1.0 [19-07-18]

You can choose whether your patient information is used for research at [name of clinic].

How your data is used

Your health and care information is used to improve your individual care. It is also used to help us research new treatments and plan your local services. We only use *anonymised* data for research studies – this is information which does not identify you.

Who can use your patient information for research and planning?

It is used by the NHS, local authorities, university and hospital researchers, medical colleges and pharmaceutical companies researching new treatments.

Making your data opt-out choice

You can choose to opt out of sharing your anonymised patient information for research at [name of clinic]. There may still be times when your patient information is used: for example, during an epidemic where there might be a risk to your or to other people's health. You can also still consent to take part in a specific research project.

Will choosing this opt-out affect your care and treatment?

No, your patient information will still be used for your individual care. Choosing to opt out will not affect your care and treatment.

What should I do next?

You do not need to do anything if you are happy about how your patient information is used at [name of clinic].

If you do not want your anonymised patient information to be used for research at [name of clinic], you can choose to opt out. Please tell our reception staff.

You can change your choice at any time.

To find out more, please contact: [name of clinical lead at clinic]
