# Supplementary material for: Accelerated partner therapy (APT) partner notification for people with Chlamydia trachomatis: protocol for the Limiting Undetected Sexually Transmitted infections to RedUce Morbidity (LUSTRUM) APT cross-over cluster randomised controlled trial
Source: BMJ Open. 2020 Mar 29;10(3):e034806. doi: 10.1136/bmjopen-2019-034806 (PMC7170609; doi:10.1136/bmjopen-2019-034806)
Supplement: Supplementary data [file bmjopen-2019-034806supp002.pdf]

[INSERT SITE LOGO]

Appendix E

PE Studies 1 & 2

Title of the project: Accelerated Partner Therapy (APT) Process Evaluation Study
Centre Number:
Study Number:

VERBAL CONSENT FORM
(Sexual Health clinic patients and sex partners)

Name of Chief Investigator: Prof. Claudia Estcourt

Researcher to initial boxes

1. I confirm that I have read and understood the information sheet dated 22-06-18, version 2.1 for the above study. I have had the opportunity to consider the information, ask questions and I am satisfied with the answers. ☐
2. I am aged between 16 and 65 years of age. ☐
3. I understand that my participation is voluntary and that I am free to withdraw from the study at any time without giving a reason, without my medical care or legal rights being affected. ☐
4. I understand that relevant sections of my demographic information and data collected during the LUSTRUM Accelerated Partner Therapy (APT) Chlamydia Partner Notification Trial may be looked at by researchers from Glasgow Caledonian University and University College London, where it is relevant to my taking part in this study. I give permission for these individuals to have access to my records. ☐
5. I understand that the information collected about me will be used to support other research in the future, and may be shared anonymously with other researchers. ☐
6. I understand that if I or someone else is believed to be in danger or at risk of significant harm, this will be reported directly to [name], [Lead for safeguarding] and Professor Claudia Estcourt, Chief Investigator on the study. Any allegations of poor practice discovered during the study will be reported directly to [name], Head of Service for GU/HIV medicine and [name], Service Manager. ☐
7. I give permission for the interview to be audio-recorded and transcribed. ☐
8. I agree to the use of direct quotations from my interview in publications, reports and/or presentations provided that anonymity is preserved. I understand that the results of this study may be published; however my anonymity will be preserved. ☐
9. I agree to take part in the above study. ☐
10. I confirm that I am still happy for my data to be included in the study (**to be completed at the end of the session**). ☐

____________________	____________________	____________________
Name of participant	Date	Time verbal consent obtained
____________________	____________________	____________________
Name of person taking consent	Date	Signature

Central and North West London NHS
NHS Foundation Trust

LUSTRUM
Limiting Undetected Sexually Transmitted Infections to RedUce Morbidity

[INSERT SITE LOGO]

Interview consent form (Patients & Sex Partners) - Process Evaluation Studies 1&2
Version 2.1 [22-06-18]
IRAS 245000

When completed: 1 for researcher site file.

Central and North West London NHS
NHS Foundation Trust

LUSTRUM
Limiting Undetected Sexually Transmitted Infections to RedUce Morbidity
