# Supplementary material for: Accelerated partner therapy (APT) partner notification for people with Chlamydia trachomatis: protocol for the Limiting Undetected Sexually Transmitted infections to RedUce Morbidity (LUSTRUM) APT cross-over cluster randomised controlled trial
Source: BMJ Open. 2020 Mar 29;10(3):e034806. doi: 10.1136/bmjopen-2019-034806 (PMC7170609; doi:10.1136/bmjopen-2019-034806)
Supplement: Supplementary data [file bmjopen-2019-034806supp004.pdf]

[INSERT SITE LOGO]

Interview consent form (Healthcare Professionals)
Process Evaluation Study 3
Version 2.1 [22-06-18]
IRAS 000245

Appendix E

PE Study 3

Title of the project: Accelerated Partner Therapy (APT) Process Evaluation Study

Centre Number:

Study Number:

CONSENT FORM

(Healthcare Professionals – Interviews)

Name of Chief Investigator: Prof. Claudia Estcourt

Participant to initial boxes

1. I confirm that I have read and understood the information sheet dated 22-06-18, Version 2.1 for the above study. I have had the opportunity to consider the information, ask questions and I am satisfied with the answers. ☐
2. I understand that my participation is voluntary and that I am free to withdraw from the study at any time without giving a reason. ☐
3. I understand that the information collected about me will be used to support other research in the future, and may be shared anonymously with other researchers. ☐
4. I understand that if I or someone else is believed to be in danger or at risk of significant harm, this will be reported directly to [name], [Lead for safeguarding] and Professor Claudia Estcourt, Chief Investigator on the study. Any allegations of poor practice discovered during the study will be reported directly to [name], Head of Service for GU/HIV medicine and [name], Service Manager. ☐
5. I give permission for the interview discussion to be audio-recorded and transcribed ☐
6. I agree to the use of direct quotations from the interview in publications, reports and/or presentations provided that anonymity is preserved. I understand that the results of this study may be published; however my anonymity will be preserved. ☐
7. I agree to take part in the above study. ☐
8. I confirm that I am still happy for my data to be included in the study **(to be completed at the end of the session).** ☐

____________________	____________________	____________________
Name of participant	Date	Signature

Central and North West London NHS
NHS Foundation Trust

LUSTRUM
Limiting Undetected Sexually Transmitted Infections to RedUce Morbidity

[INSERT SITE LOGO]

Interview consent form (Healthcare Professionals)
Process Evaluation Study 3
Version 2.1 [22-06-18]
IRAS 000245

______________________	______________________	______________________
Name of person taking consent	Date	Signature

When completed: 1 for participant; 1 for researcher site file.

Central and North West London NHS
NHS Foundation Trust

LUSTRUM
Limiting Undetected Sexually Transmitted Infections to RedUce Morbidity
