# Supplementary material for: Accelerated partner therapy (APT) partner notification for people with Chlamydia trachomatis: protocol for the Limiting Undetected Sexually Transmitted infections to RedUce Morbidity (LUSTRUM) APT cross-over cluster randomised controlled trial
Source: BMJ Open. 2020 Mar 29;10(3):e034806. doi: 10.1136/bmjopen-2019-034806 (PMC7170609; doi:10.1136/bmjopen-2019-034806)
Supplement: Author's manuscript [file bmjopen-2019-034806.draft_revisions.pdf]

BMJ Open

BMJ Open is committed to open peer review. As part of this commitment we make the peer review history of every article we publish publicly available.

When an article is published we post the peer reviewers' comments and the authors' responses online. We also post the versions of the paper that were used during peer review. These are the versions that the peer review comments apply to.

The versions of the paper that follow are the versions that were submitted during the peer review process. They are not the versions of record or the final published versions. They should not be cited or distributed as the published version of this manuscript.

BMJ Open is an open access journal and the full, final, typeset and author-corrected version of record of the manuscript is available on our site with no access controls, subscription charges or pay-per-view fees (http://bmjopen.bmj.com).

If you have any questions on BMJ Open's open peer review process please email info.bmjopen@bmj.com

BMJ Open

Accelerated Partner Therapy (APT) partner notification for people with Chlamydia trachomatis: protocol for the Limiting Undetected Sexually Transmitted infections to Reduce Morbidity (LUSTRUM) APT cross-over cluster randomised controlled trial

Journal:	*BMJ Open*
Manuscript ID	bmjopen-2019-034806
Article Type:	Protocol
Date Submitted by the Author:	08-Oct-2019

Complete List of Authors:	Estcourt, Claudia; Glasgow Caledonian University, SHLS<br>Howarth, Alison; UCL, Institute for Global health<br>Copas, Andrew; UCL, Institute for Global Health<br>Low, Nicola; University of Bern, Bern, Switzerland, Institute of Social and Preventive Medicine<br>Mapp, Fiona; UCL, Institute for Global Health<br>Woode Owusu, Melvina; UCL, Institute for Global Health<br>Flowers, Paul; University of Glasgow, Institute of Health and Wellbeing<br>Roberts, Tracy; University of Birmingham, Health Economics Unit<br>Mercer, Catherine; University College London, Institute for Global Health<br>Wayal, Sonali; Development Media International Cic; UCL, Institute for Global Health<br>Symonds, Merle; Western Sussex Hospitals NHS Foundation Trust<br>Nandwani, Rak; NHS Greater Glasgow & Clyde,<br>Saunders, John; Public Health England; University College London, Institute for Global Health<br>Johnson, Anne; University College London, Institute for Global Health<br>Pothoulaki, Maria; Glasgow Caledonian University<br>Althaus, Christian; University of Bern, Institute of Social and Preventive Medicine<br>Pickering, Karen; University of Birmingham, Health Economics Unit<br>McKinnon, Tamsin; UCL, Institute for Global Health<br>Brice, Susannah; Barts Health NHS Trust<br>Comer, Alex; Barts Health NHS Trust<br>Tostevin, Anna; UCL, Centre for Clinical Research in Infection and Sexual Health, Institute for Global Health<br>Ogwulu , Chidubem (Duby); University of Birmingham, Health Economics Unit<br>Vojt, Gabriele; Glasgow Caledonian University<br>Cassell, Jackie; Brighton and Sussex Medical School, Department of Sexually Transmitted Diseases

Keywords:	Partner notification, STIs, chlamydia, accelerated partner therapy, RCT, transmission

I, the Submitting Author has the right to grant and does grant on behalf of all authors of the Work (as defined in the below author licence), an exclusive licence and/or a non-exclusive licence for contributions from authors who are: i) UK Crown employees; ii) where BMJ has agreed a CC-BY licence shall apply, and/or iii) in accordance with the terms applicable for US Federal Government officers or employees acting as part of their official duties; on a worldwide, perpetual, irrevocable, royalty-free basis to BMJ Publishing Group Ltd ("BMJ") its licensees and where the relevant Journal is co-owned by BMJ to the co-owners of the Journal, to publish the Work in this journal and any other BMJ products and to exploit all rights, as set out in our licence.

The Submitting Author accepts and understands that any supply made under these terms is made by BMJ to the Submitting Author unless you are acting as an employee on behalf of your employer or a postgraduate student of an affiliated institution which is paying any applicable article publishing charge ("APC") for Open Access articles. Where the Submitting Author wishes to make the Work available on an Open Access basis (and intends to pay the relevant APC), the terms of reuse of such Open Access shall be governed by a Creative Commons licence – details of these licences and which Creative Commons licence will apply to this Work are set out in our licence referred to above.

Other than as permitted in any relevant BMJ Author's Self Archiving Policies, I confirm this Work has not been accepted for publication elsewhere, is not being considered for publication elsewhere and does not duplicate material already published. I confirm all authors consent to publication of this Work and authorise the granting of this licence.

For peer review only

Accelerated Partner Therapy (APT) partner notification for people with *Chlamydia trachomatis*: protocol for the Limiting Undetected Sexually Transmitted infections to Reduce Morbidity (LUSTRUM) APT cross-over cluster randomised controlled trial

Authors: Claudia S Estcourt, Alison R Howarth, Andrew Copas, Nicola Low, Fiona Mapp, Melvina Wood Owusu, Paul Flowers, Tracy Roberts, Catherine H Mercer, Sonali Wayal, Merle Symonds, Rak Nandwani, John Saunders, Anne M Johnson, Maria Pothoulaki, Christian Althaus, Karen Pickering, Tamsin McKinnon, Susannah Brice, Alex Comer, Anna Tostevin, Chidubem Ogwulu (Duby), Gabriele Vojt, Jacqueline Cassell

Corresponding author:

Prof Claudia S Estcourt
Postal address: Room 421, George Moore Building, Glasgow Caledonian University, Glasgow, G4 0BA
Email address: Claudia.estcourt@gcu.ac.uk
Telephone number: 0141 331 3099

Word count: 4525

For peer review only

For peer review only - http://bmjopen.bmj.com/site/about/guidelines.xhtml

ABSTRACT

Introduction: Partner notification (PN) is a process aiming to identify, test and treat the sex partners of people (index patients) with sexually transmitted infections (STI). Accelerated Partner Therapy (APT) is a PN method whereby healthcare professionals assess sex partners, by telephone consultation, before giving the index patient antibiotics and STI self-sampling kits to deliver to their sex partner(s). The Limiting Undetected Sexually Transmitted infections to RedUce Morbidity (LUSTRUM) programme aims to determine the effectiveness of APT in heterosexual women and men with chlamydia and determine whether APT could affect *Chlamydia trachomatis* transmission at population level.

Methods and analysis: This protocol describes a cross-over cluster randomised controlled trial of APT, offered as an additional PN method, compared with standard PN. The trial is accompanied by an economic evaluation, transmission dynamic modelling, and a qualitative process evaluation involving patients, partners and healthcare professionals. Clusters are 17 sexual health clinics in areas of England and Scotland with contrasting patient demographics. We will recruit 5440 heterosexual women and men with chlamydia, aged ≥16 years.

The primary outcome is the proportion of index patients testing positive for *C. trachomatis* 12-16 weeks after the PN consultation. Secondary outcomes include: proportion of sex partners treated; cost-effectiveness; model-predicted chlamydia prevalence; experiences of APT.

The primary outcome analysis will be by intention-to-treat, fitting random effects logistic regression models that account for clustering of index patients within clinics and trial periods. The transmission dynamic model will be used to predict change in chlamydia prevalence following APT. The economic evaluation will use mathematical modelling outputs, taking a health service perspective. Qualitative data will be analysed using interpretative phenomenological analysis and framework analysis.

Ethics and dissemination: This protocol received ethical approval from London - Chelsea Research Ethics Committee (18/LO/0773). Findings will be published with open access licences.

Trial registration: ISRCTN Reference 15996256

Key words: Partner notification; STIs; chlamydia; accelerated partner therapy; RCT; transmission

For peer review only

For peer review only - http://bmjopen.bmj.com/site/about/guidelines.xhtml

STRENGTHS AND LIMITATIONS OF THIS STUDY

Article Summary:

- The trial will evaluate APT, a novel approach to partner notification (PN), designed to facilitate and accelerate testing and treatment of sex partners of people with chlamydia
- The primary outcome, chlamydia positivity, is objectively measured and clinically relevant, given its negative impact on both individual health outcomes and NHS costs
- This clinic-wide low-risk intervention has been granted ethical approval at the service-level without need for individual consent and complies with GDPR
- The pragmatic trial design will ensure that the effectiveness of APT is evaluated under real-life clinical conditions
- An integral process evaluation will capture any variations in the operationalisation of PN and APT at the local level and enable optimisation of APT procedures in any future roll-out

INTRODUCTION

Partner notification (PN) is the process of identifying, testing and treating sex partners of a person with a sexually transmitted infection (STI).(1) PN is a key element of STI control on several levels.(2) It should benefit the individual diagnosed with the STI (the index patient) by preventing re-infection, the sex partner who might be the source of infection or could transmit undiagnosed infections to new sexual partners, and it should help to reduce spread of STIs in sexual networks and populations.

STIs are a major public health concern. The sexually transmitted pathogen *Chlamydia trachomatis* causes chlamydia infection, the most commonly reported bacterial STI in Britain,(3–5) with 218,095 diagnosed cases in England in 2018.(3) Untreated chlamydial infection can lead to pelvic inflammatory disease (PID), infertility, ectopic pregnancy and chronic pelvic pain in women.(6) In prospective studies with active follow up, about 20% of women have a repeat diagnosis of chlamydia infection in the year after treatment.(7–9)

Chlamydia screening programmes aim to reduce the occurrence of complications by detecting and treating infections. For example the incidence of diagnosed chlamydia in England has increased because of increases in both testing and transmission.(3,10–12) Chlamydia control activities in England include a national chlamydia screening programme (NCSP), which offers chlamydia testing to sexually active adults aged under 25 years, who accounted for 61% of diagnosed chlamydia infections in 2018.(13) The Public Health Outcomes Framework in England sets targets to increase chlamydia diagnosis in young men and women.(14) However a mathematical modelling study suggested that improving PN outcomes for chlamydia would be more cost-effective than increasing

For peer review only - http://bmjopen.bmj.com/site/about/guidelines.xhtml

the coverage of chlamydia testing,(2) emphasizing the importance of optimising the effectiveness of PN whatever the coverage of testing.

Like many other bacterial STIs, chlamydia does not induce lasting immunity after antibiotic treatment, and this represents a particular challenge for STI control efforts. Repeated infection after treatment can result from re-infection from an untreated partner, indicating failed PN, or from a new infection from a new partner. Alternatively, infection might persist if antibiotic treatment is not effective for any reason. It is usually not possible to determine the reason for the repeat positive test (re-infection, new infection or treatment failure). In a mathematical modelling study, the peak incidence of repeated infections was estimated at 2 to 5 months after treatment.(15)

Given the importance of PN in the control of STIs such as chlamydia, there is a need to optimise sexual health services' support for effective PN. Currently, healthcare professionals in British sexual health services often struggle to meet modest targets.(16–18) The PN process is challenging both for patients who may face barriers to informing sex partner(s) about the STI, and to services where trained staff need time to elicit sensitive information from patients in order to support PN.

Offering patients alternatives, so that they can choose the most acceptable PN method - which might differ between sex partners - is considered optimal practice.(19) For people with chlamydia, PN is most often performed through patient referral,(20,21) in which a healthcare professional advises the person with a diagnosed infection (the index patient) to inform their sex partner(s) of the need for testing and treatment and to refer them to a sexual health service (simple patient referral). This basic advice can be supplemented by information, in the form of written leaflets or website addresses, for the index patient to give to their partner(s) (enhanced patient referral). Expedited partner therapy (EPT) is a strategic approach to enhanced patient referral, widely adopted in the United States of America (USA), in which a healthcare professional gives the index patient antibiotics or a prescription for their partner(s).(22) A systematic review found that EPT results in lower proportions of index cases with repeated curable STIs than simple patient referral,(16) and a USA study demonstrated decreases in chlamydia positivity and gonorrhoea incidence at the population level.(23) However, EPT, as now widely practised in the USA, does not require a consultation with the sex partner and does not comply with UK prescribing guidance,(24) so cannot be implemented in the UK.

We therefore developed accelerated partner therapy (APT) as an adaptation of EPT to speed up the enhanced patient referral process.(25–27) APT complies with UK prescribing guidance since a healthcare professional assesses the appropriateness of the prescribed antibiotics for the partner. (28) In brief, the healthcare professional performs a telephone consultation with the sex partner in

For peer review only - http://bmjopen.bmj.com/site/about/guidelines.xhtml

private during the index patient's clinic attendance. If medically safe, the index patient receives an APT pack, containing antibiotics and self-sampling kits for STI and HIV to deliver to their sex partner(s), or the clinic may post the APT pack to the sex partner(s). In pilot studies, APT resulted in faster sex partner treatment and greater overall numbers of sex partners treated, when compared with standard PN, but lower levels of testing for HIV and other STIs, when offered without HIV testing as part of the pack.(25,26)

In the trial described here, we aimed to compare strategies for PN, specifically APT with routine PN approaches as currently practised in the UK. As part of the Limiting Undetected STi to RedUce Morbidity (LUSTRUM) programme (lustrum.org.uk), we designed a randomised controlled trial (RCT) to determine the effects of APT for people with *C. trachomatis* on biological, as well as patient-reported clinical outcomes. Specific objectives are to determine: 1. the effect of APT on the proportion of index patients who test positive for chlamydia 12-16 weeks after the PN consultation; 2. the effect of APT on the proportion of sex partners treated; 3. the cost-effectiveness of APT on long-term sexual and reproductive health outcomes, based on a linked transmission dynamic modelling study; 4. the effects of APT according to sex partner type; 5. whether APT is associated with faster treatment than standard PN, 6. the effects of APT on transmission of chlamydia at the population level, based on mathematical modelling; and to conduct: 7. a comprehensive process evaluation to understand the experiences of healthcare professionals, patients and sex partners of APT.

METHODS AND ANALYSIS

Trial design: LUSTRUM is a cross-over cluster RCT of APT offered as an additional PN method compared with standard PN alone. The APT intervention is offered at the level of the sexual health clinic, with randomisation of each clinic to either intervention or control arm in the first phase of the trial. The trial design is summarised in FIGURE 1.

FIGURE 1: Index patient and sex partner pathways during control and intervention arms

Settings: Seventeen NHS (publicly funded, free to access) specialist sexual health clinics (clusters) across England and Scotland with high volumes of positive *C. trachomatis* test results and with contrasting patient demographics. Clinics were selected from those expressing interest, based on numbers of reported chlamydia diagnoses data in the Public Health England Genitourinary Medicine Clinical Activity Dataset for STI surveillance (England) and geographical diversity (Scotland) to create three strata: London, non-London metropolitan "cities" and non-London urban "towns". A full list of study sites is included in the Acknowledgments below.

For peer review only - http://bmjopen.bmj.com/site/about/guidelines.xhtml

Eligibility criteria for index patients: Aged 16 years or older, positive test for *C. trachomatis* and/or clinical diagnosis of PID or cervicitis (women) or non-gonococcal urethritis (NGU) or epididymo-orchitis (men) and report at least one contactable sexual partner in the past six months. Index patients with PID, cervicitis, NGU and epididymo-orchitis whose test results are subsequently found to be negative for *C. trachomatis* will not be included in statistical analysis. Exclusion criteria are: groups of patients with more complex PN requirements; co-infection with other STIs including HIV, men who have had sex with men (MSM) in the past six months, and patients who have paid for or who have been paid for sex in the past six months. Patients with any clinical, social or other circumstances, such as sexual assault or insufficient English language skills to safely engage in telephone consultations, which emerge during the face-to-face consultation and make APT unsuitable will also be excluded.

Eligibility criteria for sex partners: Named as a sex partner of the index patient within the appropriate look back period (six months for chlamydia, three months for PID, one month for NGU) (29) and selected by the index patient for APT and aged 16 years or older.

Intervention: The APT intervention is a complex intervention involving index patient, healthcare professional and sex partner, situated within wider clinical care (FIGURE 1). In qualitative research studies to optimise the intervention, we explored the acceptability of individual components of the APT intervention with members of the public, sexual health clinic attenders and healthcare professionals.(30) During the intervention phase, clinics will offer APT as an additional option for eligible patients, alongside standard PN, described in BOX 1 for index patients, and BOX 2 and FIGURE 2 for sex partners. Eligible index patients who receive standard PN will receive the same follow-up as those who receive APT. If APT is no longer feasible (e.g. sex partner unavailable), standard PN will be offered instead.

For peer review only

For peer review only - http://bmjopen.bmj.com/site/about/guidelines.xhtml

BOX 1: Overview of APT for index patients

1. Index patient has PN consultation with healthcare professional (HCP); HCP assesses eligibility for APT.
2. Eligible index patient is offered APT alongside the clinic's other standard PN options. The patient can choose different methods for different partners.
3. Index patient telephones or messages sex partner (with or without the HCP present, according to preference) to offer immediate telephone assessment by the HCP.
4. Index patient waits in clinic while the HCP conducts APT telephone consultation in private with sex partner.
5. Index patient is informed that they will receive a follow-up telephone call in two weeks and they will either receive a chlamydia self-sampling postal kit in 12-16 weeks (preferred), or they may re-attend the clinic for testing.
6. Two-week follow-up: Research Health Adviser (RHA) telephones index patient to find out about PN outcomes with partner(s), to remind them of the repeat test, and to invite them to be contacted about taking part in a telephone interview regarding their experiences of APT (process evaluation).
7. 12 weeks: Index patient is sent a personalised text reminder about repeat test.
8. 13 weeks: Index patient is sent a self-sample kit by The Doctors Laboratory (TDL). Index patient returns self-collected sample or attends clinic for repeat testing. S/he receives results either via text message from TDL (negative results) or using routine clinic systems (positive or equivocal results). Positive results are managed according to routine clinic protocol. If the index patient does not return a self-sample or attend clinic for repeat testing, they receive a personalised text reminder eight days after the self-sample kit is sent out, followed by a telephone call from the RHA 13 days after the self-sample kit is sent out. Self-samples received >24 weeks post-PN interview are excluded.

BOX 2: Overview of APT for sex partners

1. Index patient telephones sex partner to inform them about exposure to chlamydia and offer immediate telephone assessment (APT).
2. If sex partner agrees to APT, HCP telephones them and conducts a clinical assessment in private. If appropriate, sex partner is offered an APT pack (delivered by the index patient or mailed directly). Sex partners for whom APT is inappropriate or who do not wish to continue with the APT option, will be advised by the HCP to attend clinic for further management. During the same telephone call, HCP invites sex partner to be contacted about taking part in a telephone interview regarding their experiences of APT (process evaluation).
3. Sex partner receives APT pack (FIGURE 2), which contains: antibiotics (either azithromycin or doxycycline, depending on local clinic practice); condoms; information about chlamydia, gonorrhoea, HIV and syphilis; chlamydia and gonorrhoea self-sampling kit, HIV and syphilis self-sampling kit, and information leaflet about how to take a sample (including link to an explanatory online video: lustrum.org.uk/test-and-treat); request form for the sample to be processed by the lab; envelope for return of self-sampling kits; and APT pack packaging (envelope or small box, no branding or other identifiable markings, and which fits through standard letterbox).
5. Sex partner completes self-sampling, labels, and returns samples for testing.
6. Sex partner takes antibiotic treatment.
7. Sex partner informed of test results by text (negative results) or routine clinic processes; positive results are managed according to routine care.

For peer review only - http://bmjopen.bmj.com/site/about/guidelines.xhtml

FIGURE 2: Contents of sex partner APT pack

Control: During the control phase, each clinic follows their usual protocols for standard PN. All clinics conduct enhanced patient referral, including verbal information about notifying partners plus additional information (written leaflets or signposting to a website). The healthcare professional will occasionally notify partners on behalf of the patient, if requested. Follow-up telephone calls and repeat testing will be the same as those during the APT phase.

Outcomes: The primary clinical outcome is the proportion of index patients with a positive test result for *C. trachomatis* 12-16 weeks after the PN consultation. A repeated positive test result after treatment is a proxy for re-infection from an untreated partner, assuming that other reasons for a repeat positive test result are distributed equally between intervention and control arms. This outcome has been used in RCTs of PN interventions seeking to demonstrate prevention of transmission at the level of the individual.(16)

Secondary clinical outcomes are: 1) the proportion of sex partners treated two weeks after the initial PN consultation; 2) numbers of partners treated per index patient; 3) time to partner treatment; and 4) number of patients notified per index patient. These outcomes will be ascertained by index patient reports at the two-week follow-up telephone call with the RHA. We will explore acceptability of the APT intervention to index patients and sex partners through qualitative telephone interviews with patients and partners as part of the process evaluation. We will determine rates of STI and HIV testing in sex partners and proportions of positive test results for STIs and HIV among sex partners who return postal self-sampling kits contained in APT packs. We will determine costs associated with the intervention in economic evaluation studies based on costings of the trial. We will record any adverse events using local activity logs at each trial site and report them to the trial sponsor and ethics committee.

Sample size: The trial is designed to determine superiority of APT as an adjunct to standard PN compared with standard PN. Our calculation is based on recruiting an average of 160 index patients per clinic per trial phase from the 17 participating clinical services (total 5440 patients) and a coefficient of variation in the number recruited of 0.5. We expect that 50% of recruited patients (80 per clinic per phase and 2720 total) will contribute to the analysis of the primary outcome because we restrict this analysis to patients with *C. trachomatis* detected at baseline, we assume that 60% of index patients will provide a repeat sample for *C. trachomatis* detection and we expect that 10 to 25% of patients in the control arm will have *C. trachomatis* detected at the 12-16 weeks follow up. (16,31,32) This sample size provides 80% power (at the 5% significance level) to detect a fall in *C. trachomatis* positivity from 10% to 5%, and 82% power to detect a fall from 25 to 17%. We consider

For peer review only - http://bmjopen.bmj.com/site/about/guidelines.xhtml

such reductions would be reasonable if around half the index patients in the intervention phase select APT for some or all their partners, but our calculation does not assume any specific value of APT uptake. A period of six months in the control condition and six months in the intervention condition should be sufficient to reach this target. Related to our key secondary outcome (the proportion of sex partners treated), for simplicity, we consider power to detect an effect on the proportion of index patients with one or more partner treated. This sample size provides 87% power to detect an increase from 60% in the control arm to 70% in the intervention arm.(17)

Sample size calculations are guided by Giraudeau et al. (33) and performed as if the trial were a standard cluster RCT with 17 clinics in each arm. The cross-over design in our trial, with each cluster contributing data to both intervention and control conditions, gives more information about the intervention effect and so greater power than stated above. Our calculations assume an intra-cluster correlation (ICC) of 0.02, which is not based on published data. We believe that our power calculations are likely to be conservative because any loss from a higher ICC will be more than offset by the gain from the cross-over design.

Recruitment: During the initial PN consultation with the index patient, the healthcare professional will assess eligibility for the study for all patients with a laboratory positive test result for chlamydia or a clinical diagnosis of NGU/epididymo-orchitis (men) or PID/ cervicitis (women). During the intervention phase, the healthcare professional will offer eligible patients APT in addition to standard methods of PN. The trial data manager will monitor recruitment and patient uptake of the APT intervention throughout the trial period supplying monthly recruitment figures to the Trial Management Group (TMG) and Data Monitoring Committee (DMC).

Randomisation / allocation sequence: The random allocation of clinics, to intervention or control arm first, was conducted through random permutation within strata, using computer-generated random numbers in Stata 15 software. In four strata, the two clinics were part of one NHS trust and were considered as pairs. One stratum contained five clinics from large cities and another contained three clinics from smaller settlements. Given the odd numbers of clinics in these strata, we used random assignment to determine whether two or three clinics would implement the intervention first in the larger stratum (correspondingly two or one in the smaller stratum to ensure balance). One further clinic, the last (17th) to start recruitment was allocated through simple randomisation, giving a total of nine clinics allocated to implement the intervention first and eight to first follow routine practice. To remove the potential for allocation bias arising because allocation codes were generated after clinics had already been recruited to the trial, one statistician generated the

For peer review only - http://bmjopen.bmj.com/site/about/guidelines.xhtml

allocation codes and another randomly permuted the clinic names within the strata. Only then, a third person matched the two to reveal the allocations and inform the clinic manager.

Blinding: Blinding of allocation will not be possible for healthcare professionals, patients or research staff. A statistician blinded to allocation will replicate the analysis of the primary outcome.

Procedure for collecting data: Healthcare professionals will record their consultations in real time and download data needed for clinical purposes into the patient's clinic record. They will use RELAY, a bespoke data collection tool. RELAY is a secure web-based data collection platform, which incorporates different interfaces allowing specified levels of access to healthcare professionals, research health advisers, data manager, trial manager and the research team. It is hosted on secure servers and compliant with NHS data storage requirements. We developed RELAY for the LUSTRUM trial, based on previous versions of data collection tools in our pilot studies of APT.(25,26)

Statistical analysis: Analysis will be by intention-to-treat. For the primary, and other quantitative outcomes, we will fit random effects models with fixed effects for clinic and intervention condition, together with a random effect to acknowledge the clustering of index patients for each combination of clinic and for each period.(34) The primary and key secondary outcomes are binary, so we will use logistic regression models. The intervention effect will be expressed as an odds ratio with 95% confidence interval. Models for secondary outcomes with outcomes quantified for each sexual contact will include additional random effects for the index patient.

We anticipate loss to follow-up of around 40% of participants, raising potential concerns over bias. Before conducting our analysis of the primary outcome we will analyse predictors of follow-up from among a pre-specified list of characteristics of the index patients that will be recorded at baseline and which are considered to be likely predictors of the primary outcome. In the primary analysis of the primary outcome we will include these as covariates in our regression model along with any other key predictors of the outcome. This approach should reduce bias from loss-to-follow-up but we will also conduct further sensitivity analyses based on multiple imputation in which we will allow those lost to-follow-up to be more, and then less, likely to be chlamydia positive at three months than those not lost to follow-up with the same baseline covariates. These sensitivity analyses will follow the approach of Carpenter et al.,(35) to assess the robustness of our conclusions to different pre-specified assumptions about missing data.

Exclusions from analyses: The intervention is implemented at the clinic level and applied to all eligible patients with service-level, rather than individual, consent. Once a clinic has begun the allocated intervention then all eligible patients added to the RELAY web tool will be included in

For peer review only - http://bmjopen.bmj.com/site/about/guidelines.xhtml

analysis, following the spirit of an intention-to-treat analysis and the all-randomised population. However, in the unlikely event of a substantial period in which any clinic is subsequently unable to implement the intervention for any reason then we will also conduct an analysis that only includes patients attending the clinic while it is able to offer the intervention, in the spirit of a per-protocol analysis and a protocol compliant population. The inclusion of patients in analysis will not be influenced by whether the patient does or does not take up the offer of APT.

Patients who present with conditions managed at first attendance as presumptive urogenital chlamydia but who subsequently are found to have negative chlamydia tests will be excluded from analyses.

Trial timeline: There will be a four-month run in period (July – October 2018), consisting of rolling clinic set up including training for healthcare professionals and a period of at least two weeks of baseline data collection when healthcare professionals will use RELAY to record standard PN data. Research staff will monitor completeness of baseline data collection. The data will be used at the end of the trial to help interpret the trial findings. Then half of the clinics will enter intervention phase while the other half enter control phase, according to the randomisation schedule.

At the end of the first six-month trial phase (November 2018 – April 2019) there will be a two-week washout period where clinics will not offer APT to patients and follow their standard PN practice procedures. Clinics which start with the control phase will also observe the two-week wash-out to align the trial time periods. Then clinics cross over to the opposite arm (intervention or control) for phase two (for six months, May – November 2019). The total duration of the trial will be 19 months, allowing for a three-month follow-up period to enable outcome data collection to be completed for all patients in the second trial phase. Clinics which do not start phase one of the trial in November 2018 will complete recruitment in November 2019 and trial phases will be condensed.

Process evaluation: We will conduct an integrated process evaluation, which involves a suite of sub-studies with index patients, sex partners and healthcare professionals. In brief these studies include: 1) qualitative telephone interviews with a sample of index patients who accepted APT for one or more sex partner, which will take place up to eight weeks after their first PN consultation; 2) qualitative telephone interviews with a sample of sex partners who received APT, up to eight weeks after their APT consultation; 3) qualitative telephone interviews with healthcare professionals who delivered APT, which take place up to 16 weeks after the clinic offers APT during the intervention phase; 4) focus groups with healthcare professionals to explore the context, extent and fidelity of the intervention delivery, which will take place up to 16 weeks after the clinic offers APT during the intervention phase.

For peer review only - http://bmjopen.bmj.com/site/about/guidelines.xhtml

For peer review only

All potential participants will receive the relevant participant information sheet and the researcher will discuss this information with participants before seeking informed consent to participate. All telephone interviews will be audio-recorded with informed verbal consent prior to participation, transcribed verbatim and analysed using interpretative phenomenological analysis (36) to understand lived experiences of using APT. The expected duration of telephone interviews is 30-60 minutes. Focus groups will be audio-recorded with informed written consent prior to participation, transcribed verbatim and data will be analysed using the Framework approach.(37) Data collected will assess intervention fidelity, healthcare professionals' APT training (the behaviour change techniques they received), and their experience of APT delivery (the behaviour change techniques they themselves delivered) in relation to a range of feasibility issues such as impact upon clinic flow and wider systemic and cultural issues.

Health economics evaluation: The aim of the economic evaluation is to determine the relative cost effectiveness of APT compared with standard PN. If APT reduces the number of re-infected cases, there will be cost implications for the healthcare sector and for society as a whole because of effects on the incidence of complications. Cost effectiveness will be determined in two ways using two separate economic analyses which will explore the costs and consequences of the alternative interventions and report results in terms of Incremental Cost Effectiveness Ratios (ICERs) if applicable. The two analyses are: (i) within trial economic evaluation which will report the terms of natural units including *cost per case of re-infection avoided* (ii) model-based economic evaluation which will report results in terms of cost per QALY based on new data from recent research.(38)

The within trial evaluation will use only data collected within the trial and will draw upon data collected through provision of APT up until four months post-intervention when the index cases are re-tested. The evaluation will adopt the perspective of the NHS and will be based on an outcome of cost per case of re-infection avoided. It is acknowledged that this is an intermediate outcome. We will build on the experience of exploring the cost effectiveness of alternative versions of APT in previous exploratory and pilot studies.(25,26)

The trial will not capture the effects of APT on the transmission of chlamydia, or on female reproductive tract complications, such as PID. As for previous economic evaluations of chlamydia screening interventions,(39) we will develop a dynamic model of the epidemiology of *C. trachomatis* transmission, the APT intervention and its outcomes, which will use data from the trial. The model based economic evaluation and deterministic transmission dynamic model upon which the economic evaluation will be based, will be reported in detail in a separate publication.

For peer review only - http://bmjopen.bmj.com/site/about/guidelines.xhtml

Trial monitoring: The Trial Management Group (TMG) is responsible for the day-to-day design, delivery, and management of the trial.

The Trial Steering Committee (TSC) is an executive and independent body, providing overall supervision of the trial and ensuring that it is being conducted in accordance with the principles of Good Clinical Practice and the relevant regulations. The TSC will approve the trial protocol and any protocol amendments and provide advice to investigators on all aspects of the trial. The TSC will monitor trial progress including recruitment, data completeness, and losses to follow-up and ensure that there are no major and/or unexplained deviations from the trial protocol.

The Data Monitoring Committee (DMC) will be responsible for reviewing interim trial data and reporting back to the sponsor on the future management of the trial. The DMC will use data accumulated throughout the trial to inform guidance and recommendations for the continued ethical conduct of the trial. The APT intervention is a short, one-off intervention provided to patients as an additional PN option which complements existing standard care practices. As such, the risk of APT to patients is minimal. We will not conduct a formal interim analysis.

We will collect reports of adverse events relating to either the intervention or participation in the trial, which will be reviewed by the TSC and reported to the sponsor and ethics committee as appropriate. The TSC could recommend the trial be stopped if members are sufficiently concerned about these events. The trial will not be stopped early due to demonstration of efficacy, harm or futility in the primary outcome.

Patient and public involvement: The LUSTRUM PPI Group consists of 27 lay people, with a broad mix of demographic characteristics and a range of sexual health experiences. The group were first involved in the design of the research at the grant application stage. Their opinions guided the trial protocol and topic guides for the process evaluation interviews. The trial is conducted by qualified healthcare professionals and, as such, it is not feasible for the group to undertake any aspects of the data collection. The group strongly supported the service-level consent design which reduces the burden of participating in the research. Lay summaries of our findings will be circulated to the group to gain their perspective and feedback on our results; this may inform further analytical decisions and inputs.

ETHICS and DISSEMINATION

As this is a pragmatic cluster randomised trial, we will seek consent for trial participation from lead clinicians at participating clinics (service-level) and will not seek individual informed consent from index patients other than for the process evaluation studies. Following Weijer et al.,(40) we believe

For peer review only - http://bmjopen.bmj.com/site/about/guidelines.xhtml

that APT is a complex, “low-risk” healthcare delivery intervention. APT is offered in addition to standard PN and operationalised as a supplement to usual care, thus index patients have the choice of taking up APT or not. It is widely accepted that individual consent may not be essential in such trials,(41) in which the situation is analogous to the introduction of changed processes in routine services.(31) Notably, individual level consent is thought to have contributed to low recruitment numbers in a previous study of APT.(26) Individual informed consent will be sought from all participants in the process evaluation.

Study endpoints, whether negative or positive, will be reported and disseminated through the following channels: 1. Research findings will be published in journals with open access within 6 months of publication. 2. Research findings will be presented at UK and international meetings orally or via posters. 3. A report of the findings (in the form of the funder’s final report) will be freely available on both the funder’s website and the LUSTRUM website after publication in scientific journals. A link to the report will be circulated to stakeholders, collaborators and participating organisations, together with lay summaries of the study on the LUSTRUM website. 4. We will make presentations to community groups and clinic user groups as appropriate. 5. We will make continued use of social media channels to report findings after publication in scientific journals, in order to increase the “reach” of our findings to the public. 6. The anonymised participant level dataset, and statistical code for generating the results will be archived in a data repository after publication of the final report.

For peer review only

For peer review only - http://bmjopen.bmj.com/site/about/guidelines.xhtml

REFERENCES

1. UNAIDS. Sexually transmitted diseases: policies and principles for prevention and care. Geneva; 1999. Available from: https://www.unaids.org/en/resources/documents/1999/19990519_una97-6_en.pdf
2. Althaus CL, Turner KM, Mercer CH, Auguste P, Roberts TE, Bell G, et al. Effectiveness and cost-effectiveness of traditional and new partner notification technologies for curable sexually transmitted infections: observational study, systematic reviews and mathematical modelling. Health Technol Assess (Rockv). 2014 Jan;18(2):1–100, vii–viii.
3. Public Health England. Table 1: STI diagnoses and rates in England by gender, 2009 to 2018. London; 2019. Available from: https://www.gov.uk/government/statistics/sexually-transmitted-infections-stis-annual-data-tables
4. Health Protection Scotland. Surveillance report Genital chlamydia and gonorrhoea infection in Scotland: laboratory diagnoses 2009-2018. Health Protection Scotland; 2019. Available from: https://www.hps.scot.nhs.uk/web-resources-container/genital-chlamydia-and-gonorrhoea-infection-in-scotland-laboratory-diagnoses-2009-2018
5. Public Health Wales. HIV and STI trends in Wales - Surveillance Report [Internet]. Public Health Wales; 2018. Available from: http://www.wales.nhs.uk/sitesplus/documents/888/HIV and STI trends in Wales Report 2018_2017 -Surveillance report.pdf
6. Holmes KK, Sparling FP, Stamm WE, Piot P, Wasserheit JN, Corey L, et al., editors. Sexually Transmitted Diseases. Fourth Edi. New York, London: McGraw-Hill Medical; 2008.
7. Hosenfeld CB, Workowski KA, Berman S, Zaidi A, Dyson J, Mosure D, et al. Repeat infection with chlamydia and gonorrhea among females: A systematic review of the literature. Sex Transm Dis. 2009 Aug;36(8):478–89.
8. Scott Lamontagne D, Baster K, Emmett L, Nichols T, Randall S, McLean L, et al. Incidence and reinfection rates of genital chlamydial infection among women aged 16-24 years attending general practice, family planning and genitourinary medicine clinics in England: a prospective cohort study by the Chlamydia Recall Study Advisory Group. Sex Transm Infect. 2007 Jul;83(4):292–303.
9. Walker J, Tabrizi SN, Fairley CK, Chen MY, Bradshaw CS, Twin J, et al. Chlamydia trachomatis incidence and re-infection among young women - behavioural and microbiological characteristics. PLoS One. 2012 May 25;7(5).
10. Public Health England. Sexually Transmitted Infections and Chlamydia screening in England: 2018. London, England; 2019. Available from: https://assets.publishing.service.gov.uk/government/uploads/system/uploads/attachment_data/file/806118/hpr1919_stis-ncsp_ann18.pdf
11. Health Protection Scotland. Surveillance report Genital chlamydia and gonorrhoea infection in Scotland: laboratory diagnoses 2009-2018. Health Protection Scotland; 2019.
12. Public Health Wales. HIV and STI trends in Wales - Surveillance Report. Public Health Wales; 2018.
13. Public Health England. Table 2 (a - 1): Number of chlamydia diagnoses in England by gender, sexual risk & age group, 2014 - 2018. London, England; 2019. Available from: https://www.gov.uk/government/statistics/sexually-transmitted-infections-stis-annual-data-tables
14. Department of Health. Improving outcomes and supporting transparency Part 2: summary technical specifications of public health indicators. In: Public Health Outcomes Framework 2016 to 2019. 2017.
15. Heijne JCM, Herzog SA, Althaus CL, Tao G, Kent CK, Low N. Insights into the timing of repeated testing after treatment for Chlamydia trachomatis: data and modelling study. Sex Transm Infect. 2013 Feb;89(1):57–62.

For peer review only - http://bmjopen.bmj.com/site/about/guidelines.xhtml

16. Ferreira A, Young T, Mathews C, Zunza M, Low N. Strategies for partner notification for sexually transmitted infections, including HIV. In: Low N, editor. Cochrane Database of Systematic Reviews [Internet]. Chichester, UK: John Wiley & Sons, Ltd; 2013.
17. McClean H, Carne CA, Sullivan AK, Radcliffe KW, Ahmed-Jushuf I, National Audit Group of British Association for Sexual Health and HIV. Chlamydial partner notification in the British Association for Sexual Health and HIV (BASHH) 2011 UK national audit against the BASHH Medical Foundation for AIDS and Sexual Health Sexually Transmitted Infections Management Standards. Int J STD AIDS. 2012 Oct;23(10):748–52.
18. Healthcare Improvement Scotland. Improving Sexual Health Services in Scotland: Integration and Innovation National Overview. 2011.
19. Trelle S, Shang A, Nartey L, Cassell J a, Low N. Improved effectiveness of partner notification for patients with sexually transmitted infections: systematic review. BMJ. 2007;334(7589):354.
20. Fontaine J, Sfetcu O, Van De Laar M, Martin-Hilber A, Röllin A, Redmond S, et al. Public health benefits of partner notification for sexually transmitted infections and HIV.
21. Centers for Disease Control and Prevention. Chlamydia Infections Guidelines. In: 2015 STD Treatment Guidelines. Atlanta; 2015.
22. Centre for Disease Control and Prevention. Expedited partner therapy in the management of sexually transmitted diseases. Atlanta; 2006.
23. Golden MR, Kerani RP, Stenger M, Hughes JP, Aubin M, Malinski C, et al. Uptake and population-level impact of expedited partner therapy (EPT) on Chlamydia trachomatis and Neisseria gonorrhoeae: the Washington State community-level randomized trial of EPT. PLoS Med. 2015 Jan;12(1):e1001777.
24. General Medical Council. Good medical practice. London, England: GMC; 2013.
25. Estcourt C, Sutcliffe L, Cassell J, Mercer CH, Copas A, James L, et al. Can we improve partner notification rates through expedited partner therapy in the UK? Findings from an exploratory trial of Accelerated Partner Therapy (APT). Sex Transm Infect. 2012 Feb;88(1):21–6.
26. Estcourt CS, Sutcliffe LJ, Copas A, Mercer CH, Roberts TE, Jackson LJ, et al. Developing and testing accelerated partner therapy for partner notification for people with genital Chlamydia trachomatis diagnosed in primary care: A pilot randomised controlled trial. Sex Transm Infect. 2015 Dec;91(8):548–54.
27. Sutcliffe L, Brook MG, Chapman JL, Cassell JM, Estcourt CS. Is accelerated partner therapy a feasible and acceptable strategy for rapid partner notification in the UK?: A qualitative study of genitourinary medicine clinic attenders. Int J STD AIDS. 2009 Sep;20(9):603–6.
28. General Medical Council. Good practice in prescribing and managing medicines and devices. London, England: GMC; 2013.
29. Mcclean H, Radcliffe K, Sullivan A, Ahmed-Jushuf I. 2012 BASHH Statement on Partner Notification for Sexually Transmissible Infections. Int J STD AIDS. 2013;24(4).
30. Flowers P, Pothoulaki M, Woode Owusu M, Vojt G, Mapp F, Mercer CH, et al. P079 Using theory & evidence to optimise an Accelerated Partner Therapy intervention in the context of a chlamydia partner notification trial. STI HIV 2019 World Congr Vancouver. 2019;95(Suppl 1).
31. Cassell J, Young A. Why we should not seek individual informed consent for participation in health services research. J Med Ethics. 2002 Oct 1;28(5):313–7.
32. Van Der Helm JJ, Koekenbier RH, Van Rooijen MS, Schim Van Der Loeff MF, De Vries HJC. What Is the Optimal Time to Retest Patients with a Urogenital Chlamydia Infection? A Randomized Controlled Trial. Sex Transm Dis. 2018 Feb 1;45(2):132–7.
33. Giraudeau B, Ravaud P, Donner A. Sample size calculation for cluster randomized cross-over trials. Stat Med. 2008 Nov 29;27(27):5578–85.
34. Turner RM, White IR, Croudace T, PIP Study Group. Analysis of cluster randomized cross-over trial data: a comparison of methods. Stat Med. 2007 Jan 30;26(2):274–89.
35. Carpenter JR, Kenward MG, White IR. Sensitivity analysis after multiple imputation under

For peer review only - http://bmjopen.bmj.com/site/about/guidelines.xhtml

missing at random: a weighting approach. Stat Methods Med Res. 2007 Jun 2;16(3):259–75.
36. Smith JA, Flowers P, Larkin M. Interpretative phenomenological Analysis. London, Thousand Oaks, New Delhi, Singapore: Sage Publications Ltd.; 2009.
37. Ritchie J, Lewis J. Qualitative Research Practice. London: SAGE Publications; 2003.
38. Okeke Ogwulu C. Developing and piloting approaches for the valuation of outcomes associated with sexually transmitted infections [Internet]. University of Birmingham; 2018. Available from: https://ethos.bl.uk/OrderDetails.do?uin=uk.bl.ethos.760507
39. Low N, McCarthy A, Macleod J, Salisbury C, Campbell R, Roberts TE, et al. Epidemiological, social, diagnostic and economic evaluation of population screening for genital chlamydial infection. Health Technol Assess. 2007 Mar;11(8):iii–iv, ix–xii, 1-165.
40. Weijer C, Grimshaw JM, Taljaard M, Binik A, Boruch R, Brehaut JC, et al. Ethical issues posed by cluster randomized trials in health research. Trials. 2011 Apr 20;12:100.
41. Campbell MK, Weijer C, Goldstein CE, Edwards SJL. Do doctors have a duty to take part in pragmatic randomised trials? BMJ. 2017.

For peer review only

Required statements:

Trial Protocol Version 5.0 [29-07-19]

Acknowledgements: The authors would like to thank the following**:**

- **Trial Steering Committee:** Simon Barton (chair), Robbie Currie, David Crundwell, Artemis Koukounari, Lynis Lewis, Alec Miners, Emmanuel Rollings-Kamara, Rachel Shaw, Emma Thompson
- **Data Monitoring Committee:** Simon Barton, David Crundwell, Rebecca Turner, Artemis Koukounari
- **All members of the LUSTRUM Patient and Public Involvement Group**
- **Participating Centres:** Barking, Havering and Redbridge University Hospitals NHS Trust (A. Umaipalan), Barts Health NHS Trust (V. Apea), Buckinghamshire Healthcare NHS Trust (J. Sherrard), Chelsea and Westminster Hospital NHS Foundation Trust (C. Evans), Croydon Health Services NHS Trust (D. Phillips), Manchester University NHS Foundation Trust (G. Schembri), Midlands Partnership NHS Foundation Trust (J. Dhar), NHS Greater Glasgow and Clyde (R. Nandwani), Northamptonshire Healthcare NHS Foundation Trust (S. Herbert), Royal Berkshire NHS Foundation Trust (F. Chen), Royal Bournemouth and Christchurch Hospitals NHS Foundation Trust (K. Schroeder), Solent NHS Trust (R. Patel), University Hospitals Birmingham NHS Foundation Trust (J. Ross)

The authors would also like to thank epiGenesys who developed the RELAY software; Soazig Clifton (support with ethics submission); Jo Gibbs (support on RELAY development); Kay Musonda (administrative support); and the staff, management and patients involved in the study at all the participating sexual health clinics.

Authors' contributions: CSE is Chief Investigator of the study. CSE, JC, NL, TR, AnC, AJ, CHM, JS, MS, PF, RN and SW conceived the study and secured funding. They are responsible for the planning and delivery of the study. PF leads the process evaluation; TR leads on the health economics evaluation; and NL leads on the mathematical modelling. AnC is the trial statistician.

ARH, FM, MWO are responsible for coordination of the study. ARH, FM, MWO, KP, COO, SB, AlC, TM, MP, GV and AT are responsible for operationalisation of the study procedures. FM, MWO, KP, COO, MP and GV will contribute to data collection and analysis. ARH, SB and AlC will contribute to data

collection. CA and NL are responsible for the mathematical modelling. AT is responsible for trial data management and will contribute to data analysis.

All authors contributed to the development of the study design and establishment of procedures.

CSE led on preparing the manuscript. All authors critically reviewed and approved the final version.

Funding statement: This work presents independent research funded by the National Institute for Health Research (NIHR) under its Programme Grants for applied research Programme (Reference Number RP-PG-0614-20009). The views expressed are those of the author(s) and not necessarily those of the NHS, the NIHR or the Department of Health. The funders had no role in study design, collection, management, analysis, and interpretation of data; writing of the report; and the decision to submit the report for publication.

Trial Sponsor: Noclor, Central and North West London NHS Trust. The sponsor had no role in study design, collection, management, analysis, and interpretation of data; writing of the report; and the decision to submit the report for publication.

Competing interests statement: The authors have no competing interests.

Data sharing statement, Anonymised participant level dataset, and statistical code for generating the results will be archived in a data repository after publication of the final report.

Patient consent statement: Not required

Ethical approval: Ethical approval from London - Chelsea Research Ethics Committee (18/LO/0773).

For peer review only

FIGURE 1: Index patient and sex partner pathways during control and intervention arms

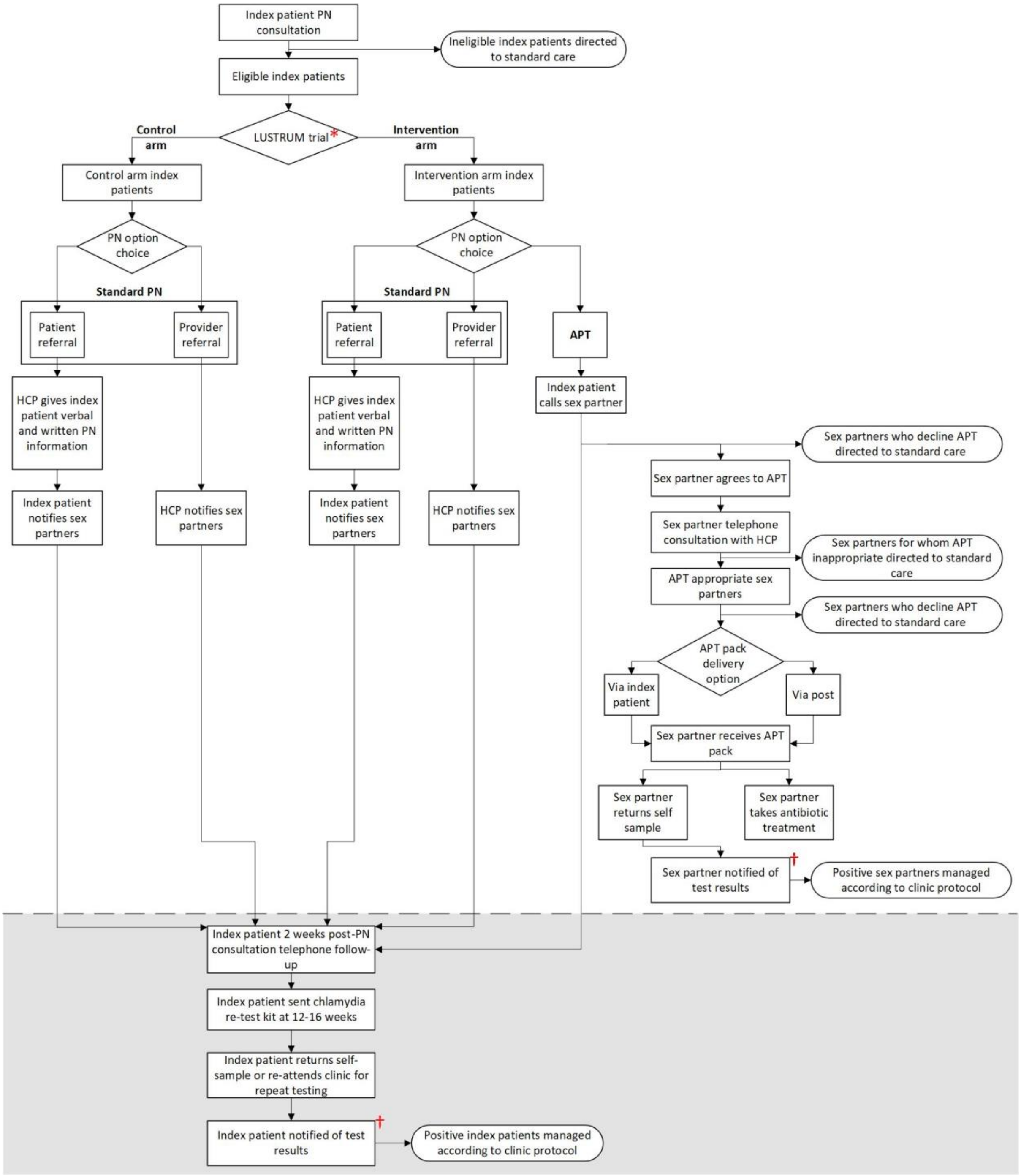

PN, Partner Notification; APT, Accelerated Partner Therapy; HCP, Healthcare Professional
* Random allocation to intervention or control arm in the first phase of the trial
† Index patients and sex partners are notified of results via two pathways: 1. Negative results: individual receives a text from The Doctors Laboratory (TDL); 2. Positive / equivocal results: individual receives results directly from clinic

FIGURE 2: Contents of sex partner APT pack

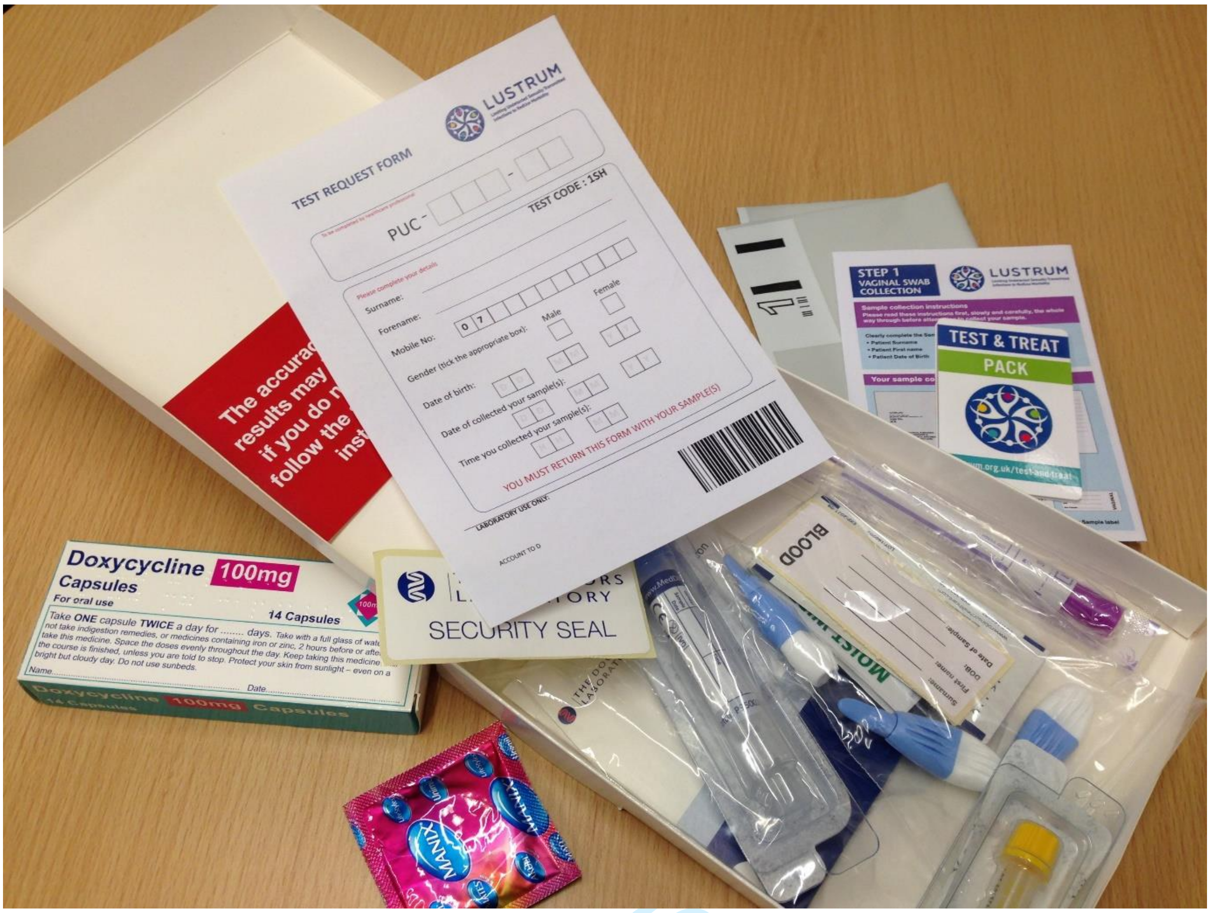

Pack contains: Antibiotics; Condoms; LUSTRUM TEST & TREAT leaflet; Vulvo-vaginal swab kit OR urine sampling kit; Blood sampling kit; Instruction leaflet for sampling kits; Test request form for sample processing; Pre-paid return post envelope; Security seal sticker; Attention card

For peer review only

SPIRIT

STANDARD PROTOCOL ITEMS: RECOMMENDATIONS FOR INTERVENTIONAL TRIALS

SPIRIT 2013 Checklist: Recommended items to address in a clinical trial protocol and related documents*

Section/item	Item No	Description	Addressed on page number
**Administrative information**			
Title	1	Descriptive title identifying the study design, population, interventions, and, if applicable, trial acronym	p1
Trial registration	2a	Trial identifier and registry name. If not yet registered, name of intended registry	p2
	2b	All items from the World Health Organization Trial Registration Data Set	Trial protocol v5.0 29.07.19
Protocol version	3	Date and version identifier	p17
Funding	4	Sources and types of financial, material, and other support	p18
Roles and responsibilities	5a	Names, affiliations, and roles of protocol contributors	p17-18
	5b	Name and contact information for the trial sponsor	p18
	5c	Role of study sponsor and funders, if any, in study design; collection, management, analysis, and interpretation of data; writing of the report; and the decision to submit the report for publication, including whether they will have ultimate authority over any of these activities	p18
	5d	Composition, roles, and responsibilities of the coordinating centre, steering committee, endpoint adjudication committee, data management team, and other individuals or groups overseeing the trial, if applicable (see Item 21a for data monitoring committee)	p13

For peer review only

**Introduction**			
Background and rationale	6a	Description of research question and justification for undertaking the trial, including summary of relevant studies (published and unpublished) examining benefits and harms for each intervention	p3-5
	6b	Explanation for choice of comparators	p4
Objectives	7	Specific objectives or hypotheses	p5
Trial design	8	Description of trial design including type of trial (eg, parallel group, crossover, factorial, single group), allocation ratio, and framework (eg, superiority, equivalence, noninferiority, exploratory)	p5
**Methods: Participants, interventions, and outcomes**			
Study setting	9	Description of study settings (eg, community clinic, academic hospital) and list of countries where data will be collected. Reference to where list of study sites can be obtained	p5
Eligibility criteria	10	Inclusion and exclusion criteria for participants. If applicable, eligibility criteria for study centres and individuals who will perform the interventions (eg, surgeons, psychotherapists)	p6
Interventions	11a	Interventions for each group with sufficient detail to allow replication, including how and when they will be administered	p6
	11b	Criteria for discontinuing or modifying allocated interventions for a given trial participant (eg, drug dose change in response to harms, participant request, or improving/worsening disease)	p7
	11c	Strategies to improve adherence to intervention protocols, and any procedures for monitoring adherence (eg, drug tablet return, laboratory tests)	p7
	11d	Relevant concomitant care and interventions that are permitted or prohibited during the trial	p6-7
Outcomes	12	Primary, secondary, and other outcomes, including the specific measurement variable (eg, systolic blood pressure), analysis metric (eg, change from baseline, final value, time to event), method of aggregation (eg, median, proportion), and time point for each outcome. Explanation of the clinical relevance of chosen efficacy and harm outcomes is strongly recommended	p8
Participant timeline	13	Time schedule of enrolment, interventions (including any run-ins and washouts), assessments, and visits for participants. A schematic diagram is highly recommended (see Figure)	p7; p11

Sample size	14	Estimated number of participants needed to achieve study objectives and how it was determined, including clinical and statistical assumptions supporting any sample size calculations	p8-9
Recruitment	15	Strategies for achieving adequate participant enrolment to reach target sample size	Not included

Methods: Assignment of interventions (for controlled trials)

Allocation:

Sequence generation	16a	Method of generating the allocation sequence (eg, computer-generated random numbers), and list of any factors for stratification. To reduce predictability of a random sequence, details of any planned restriction (eg, blocking) should be provided in a separate document that is unavailable to those who enrol participants or assign interventions	p9-10
Allocation concealment mechanism	16b	Mechanism of implementing the allocation sequence (eg, central telephone; sequentially numbered, opaque, sealed envelopes), describing any steps to conceal the sequence until interventions are assigned	p9-10
Implementation	16c	Who will generate the allocation sequence, who will enrol participants, and who will assign participants to interventions	p9-10
Blinding (masking)	17a	Who will be blinded after assignment to interventions (eg, trial participants, care providers, outcome assessors, data analysts), and how	p10
	17b	If blinded, circumstances under which unblinding is permissible, and procedure for revealing a participant's allocated intervention during the trial	N/A

Methods: Data collection, management, and analysis

Data collection methods	18a	Plans for assessment and collection of outcome, baseline, and other trial data, including any related processes to promote data quality (eg, duplicate measurements, training of assessors) and a description of study instruments (eg, questionnaires, laboratory tests) along with their reliability and validity, if known. Reference to where data collection forms can be found, if not in the protocol	p10
	18b	Plans to promote participant retention and complete follow-up, including list of any outcome data to be collected for participants who discontinue or deviate from intervention protocols	p7; p10-11

Data management	19	Plans for data entry, coding, security, and storage, including any related processes to promote data quality (eg, double data entry; range checks for data values). Reference to where details of data management procedures can be found, if not in the protocol	p10
Statistical methods	20a	Statistical methods for analysing primary and secondary outcomes. Reference to where other details of the statistical analysis plan can be found, if not in the protocol	p10-11
	20b	Methods for any additional analyses (eg, subgroup and adjusted analyses)	p11-12
	20c	Definition of analysis population relating to protocol non-adherence (eg, as randomised analysis), and any statistical methods to handle missing data (eg, multiple imputation)	p10-11
**Methods: Monitoring**			
Data monitoring	21a	Composition of data monitoring committee (DMC); summary of its role and reporting structure; statement of whether it is independent from the sponsor and competing interests; and reference to where further details about its charter can be found, if not in the protocol. Alternatively, an explanation of why a DMC is not needed	p13
	21b	Description of any interim analyses and stopping guidelines, including who will have access to these interim results and make the final decision to terminate the trial	p13
Harms	22	Plans for collecting, assessing, reporting, and managing solicited and spontaneously reported adverse events and other unintended effects of trial interventions or trial conduct	p13
Auditing	23	Frequency and procedures for auditing trial conduct, if any, and whether the process will be independent from investigators and the sponsor	Not included
**Ethics and dissemination**			
Research ethics approval	24	Plans for seeking research ethics committee/institutional review board (REC/IRB) approval	p2; p18
Protocol amendments	25	Plans for communicating important protocol modifications (eg, changes to eligibility criteria, outcomes, analyses) to relevant parties (eg, investigators, REC/IRBs, trial participants, trial registries, journals, regulators)	p13

Consent or assent	26a	Who will obtain informed consent or assent from potential trial participants or authorised surrogates, and how (see Item 32)	N/A
	26b	Additional consent provisions for collection and use of participant data and biological specimens in ancillary studies, if applicable	p12
Confidentiality	27	How personal information about potential and enrolled participants will be collected, shared, and maintained in order to protect confidentiality before, during, and after the trial	Not included
Declaration of interests	28	Financial and other competing interests for principal investigators for the overall trial and each study site	p18
Access to data	29	Statement of who will have access to the final trial dataset, and disclosure of contractual agreements that limit such access for investigators	p10
Ancillary and post-trial care	30	Provisions, if any, for ancillary and post-trial care, and for compensation to those who suffer harm from trial participation	N/A
Dissemination policy	31a	Plans for investigators and sponsor to communicate trial results to participants, healthcare professionals, the public, and other relevant groups (eg, via publication, reporting in results databases, or other data sharing arrangements), including any publication restrictions	p13-14
	31b	Authorship eligibility guidelines and any intended use of professional writers	p17-18
	31c	Plans, if any, for granting public access to the full protocol, participant-level dataset, and statistical code	p18
**Appendices**			
Informed consent materials	32	Model consent form and other related documentation given to participants and authorised surrogates	Trial protocol v5.0 29.07.19
Biological specimens	33	Plans for collection, laboratory evaluation, and storage of biological specimens for genetic or molecular analysis in the current trial and for future use in ancillary studies, if applicable	N/A

*It is strongly recommended that this checklist be read in conjunction with the SPIRIT 2013 Explanation & Elaboration for important clarification on the items. Amendments to the protocol should be tracked and dated. The SPIRIT checklist is copyrighted by the SPIRIT Group under the Creative Commons "Attribution-NonCommercial-NoDerivs 3.0 Unported" license.

Explainer paper - https://www.bmj.com/content/346/bmj.e7586.full?ijkey=QpAJnYI57zIwVr3&keytype=ref

For peer review only - http://bmjopen.bmj.com/site/about/guidelines.xhtml

BMJ Open

Accelerated Partner Therapy (APT) partner notification for people with Chlamydia trachomatis: protocol for the Limiting Undetected Sexually Transmitted infections to Reduce Morbidity (LUSTRUM) APT cross-over cluster randomised controlled trial

Journal:	*BMJ Open*
Manuscript ID	bmjopen-2019-034806.R1
Article Type:	Protocol
Date Submitted by the Author:	13-Feb-2020

Complete List of Authors:	Estcourt, Claudia; Glasgow Caledonian University, SHLS<br>Howarth, Alison; UCL, Institute for Global health<br>Copas, Andrew; UCL, Institute for Global Health<br>Low, Nicola; University of Bern, Bern, Switzerland, Institute of Social and Preventive Medicine<br>Mapp, Fiona; UCL, Institute for Global Health<br>Woode Owusu, Melvina; UCL, Institute for Global Health<br>Flowers, Paul; University of Glasgow, Institute of Health and Wellbeing<br>Roberts, Tracy; University of Birmingham, Health Economics Unit<br>Mercer, Catherine; University College London, Institute for Global Health<br>Wayal, Sonali; Development Media International Cic; UCL, Institute for Global Health<br>Symonds, Merle; Western Sussex Hospitals NHS Foundation Trust<br>Nandwani, Rak; NHS Greater Glasgow & Clyde,<br>Saunders, John; Public Health England; University College London, Institute for Global Health<br>Johnson, Anne; University College London, Institute for Global Health<br>Pothoulaki, Maria; Glasgow Caledonian University<br>Althaus, Christian; University of Bern, Institute of Social and Preventive Medicine<br>Pickering, Karen; University of Birmingham, Health Economics Unit<br>McKinnon, Tamsin; UCL, Institute for Global Health<br>Brice, Susannah; Barts Health NHS Trust<br>Comer, Alex; Barts Health NHS Trust<br>Tostevin, Anna; UCL, Centre for Clinical Research in Infection and Sexual Health, Institute for Global Health<br>Ogwulu , Chidubem (Duby); University of Birmingham, Health Economics Unit<br>Vojt, Gabriele; Glasgow Caledonian University<br>Cassell, Jackie; Brighton and Sussex Medical School, Department of Sexually Transmitted Diseases

**Primary Subject Heading**:	Sexual health
Secondary Subject Heading:	Health services research, Public health

Keywords:	Partner notification, STIs, chlamydia, accelerated partner therapy, RCT, transmission

SCHOLARONE™
Manuscripts

I, the Submitting Author has the right to grant and does grant on behalf of all authors of the Work (as defined in the below author licence), an exclusive licence and/or a non-exclusive licence for contributions from authors who are: i) UK Crown employees; ii) where BMJ has agreed a CC-BY licence shall apply, and/or iii) in accordance with the terms applicable for US Federal Government officers or employees acting as part of their official duties; on a worldwide, perpetual, irrevocable, royalty-free basis to BMJ Publishing Group Ltd ("BMJ") its licensees and where the relevant Journal is co-owned by BMJ to the co-owners of the Journal, to publish the Work in this journal and any other BMJ products and to exploit all rights, as set out in our licence.

The Submitting Author accepts and understands that any supply made under these terms is made by BMJ to the Submitting Author unless you are acting as an employee on behalf of your employer or a postgraduate student of an affiliated institution which is paying any applicable article publishing charge ("APC") for Open Access articles. Where the Submitting Author wishes to make the Work available on an Open Access basis (and intends to pay the relevant APC), the terms of reuse of such Open Access shall be governed by a Creative Commons licence – details of these licences and which Creative Commons licence will apply to this Work are set out in our licence referred to above.

Other than as permitted in any relevant BMJ Author's Self Archiving Policies, I confirm this Work has not been accepted for publication elsewhere, is not being considered for publication elsewhere and does not duplicate material already published. I confirm all authors consent to publication of this Work and authorise the granting of this licence.

For peer review only

Accelerated Partner Therapy (APT) partner notification for people with *Chlamydia trachomatis*: protocol for the Limiting Undetected Sexually Transmitted infections to Reduce Morbidity (LUSTRUM) APT cross-over cluster randomised controlled trial

Authors: Claudia S Estcourt, Alison R Howarth, Andrew Copas, Nicola Low, Fiona Mapp, Melvina Woode Owusu, Paul Flowers, Tracy Roberts, Catherine H Mercer, Sonali Wayal, Merle Symonds, Rak Nandwani, John Saunders, Anne M Johnson, Maria Pothoulaki, Christian Althaus, Karen Pickering, Tamsin McKinnon, Susannah Brice, Alex Comer, Anna Tostevin, Chidubem Ogwulu (Duby), Gabriele Vojt, Jacqueline Cassell

Corresponding author:

Prof Claudia S Estcourt

Postal address: Room 421, George Moore Building, Glasgow Caledonian University, Glasgow, G4 0BA

Email address: Claudia.estcourt@gcu.ac.uk

Telephone number: 0141 331 3099

Word count: 4580

For peer review only

ABSTRACT

Introduction: Partner notification (PN) is a process aiming to identify, test and treat the sex partners of people (index patients) with sexually transmitted infections (STI). Accelerated Partner Therapy (APT) is a PN method whereby healthcare professionals assess sex partners, by telephone consultation, before giving the index patient antibiotics and STI self-sampling kits to deliver to their sex partner(s). The Limiting Undetected Sexually Transmitted infections to RedUce Morbidity (LUSTRUM) programme aims to determine the effectiveness of APT in heterosexual women and men with chlamydia and determine whether APT could affect *Chlamydia trachomatis* transmission at population level.

Methods and analysis: This protocol describes a cross-over cluster randomised controlled trial of APT, offered as an additional PN method, compared with standard PN. The trial is accompanied by an economic evaluation, transmission dynamic modelling, and a qualitative process evaluation involving patients, partners and healthcare professionals. Clusters are 17 sexual health clinics in areas of England and Scotland with contrasting patient demographics. We will recruit 5440 heterosexual women and men with chlamydia, aged ≥16 years.

The primary outcome is the proportion of index patients testing positive for *C. trachomatis* 12-16 weeks after the PN consultation. Secondary outcomes include: proportion of sex partners treated; cost-effectiveness; model-predicted chlamydia prevalence; experiences of APT.

The primary outcome analysis will be by intention-to-treat, fitting random effects logistic regression models that account for clustering of index patients within clinics and trial periods. The transmission dynamic model will be used to predict change in chlamydia prevalence following APT. The economic evaluation will use mathematical modelling outputs, taking a health service perspective. Qualitative data will be analysed using interpretative phenomenological analysis and framework analysis.

Ethics and dissemination: This protocol received ethical approval from London - Chelsea Research Ethics Committee (18/LO/0773). Findings will be published with open access licences.

Trial registration: ISRCTN Reference 15996256

Key words: Partner notification; STIs; chlamydia; accelerated partner therapy; RCT; transmission

For peer review only

STRENGTHS AND LIMITATIONS OF THIS STUDY

Article Summary:

- The trial will evaluate APT, a novel approach to partner notification (PN), designed to facilitate and accelerate testing and treatment of sex partners of people with chlamydia
- The primary outcome, chlamydia positivity, is objectively measured and clinically relevant, given its negative impact on both individual health outcomes and NHS costs
- This clinic-wide low-risk intervention has been granted ethical approval at the service-level without need for individual consent and complies with GDPR
- The pragmatic trial design will ensure that the effectiveness of APT is evaluated under real-life clinical conditions
- An integral process evaluation will capture any variations in the operationalisation of PN and APT at the local level and enable optimisation of APT procedures in any future roll-out

INTRODUCTION

Partner notification (PN) is the process of identifying, testing and treating sex partners of a person with a sexually transmitted infection (STI).(1) PN is a key element of STI control on several levels.(2) It should benefit the individual diagnosed with the STI (the index patient) by preventing re-infection, the sex partner who might be the source of infection or could transmit undiagnosed infections to new sexual partners, and it should help to reduce spread of STIs in sexual networks and populations.

STIs are a major public health concern. The sexually transmitted pathogen *Chlamydia trachomatis* causes chlamydia infection, the most commonly reported bacterial STI in Britain,(3–5) with 218,095 diagnosed cases in England in 2018.(3) Untreated chlamydial infection can lead to pelvic inflammatory disease (PID), infertility, ectopic pregnancy and chronic pelvic pain in women.(6) In prospective studies with active follow up, about 20% of women have a repeat diagnosis of chlamydia infection in the year after treatment.(7–9)

Chlamydia screening programmes aim to reduce the occurrence of complications by detecting and treating infections. For example the incidence of diagnosed chlamydia in England has increased because of increases in both testing and transmission.(3,10–12) Chlamydia control activities in England include a national chlamydia screening programme (NCSP), which offers chlamydia testing to sexually active adults aged under 25 years, who accounted for 61% of diagnosed chlamydia infections in 2018.(13) The Public Health Outcomes Framework in England sets targets to increase chlamydia diagnosis in young men and women.(14) However a mathematical modelling study suggested that improving PN outcomes for chlamydia would be more cost-effective than increasing

For peer review only - http://bmjopen.bmj.com/site/about/guidelines.xhtml

the coverage of chlamydia testing,(2) emphasizing the importance of optimising the effectiveness of PN whatever the coverage of testing.

Like many other bacterial STIs, chlamydia does not induce lasting immunity after antibiotic treatment, and this represents a particular challenge for STI control efforts. Repeated infection after treatment can result from re-infection from an untreated partner, indicating failed PN, or from a new infection from a new partner. Alternatively, infection might persist if antibiotic treatment is not effective for any reason. It is usually not possible to determine the reason for the repeat positive test (re-infection, new infection or treatment failure). In a mathematical modelling study, the peak incidence of repeated infections was estimated at 2 to 5 months after treatment.(15)

Given the importance of PN in the control of STIs such as chlamydia, there is a need to optimise sexual health services' support for effective PN. Currently, healthcare professionals in British sexual health services often struggle to meet modest targets.(16–18) The PN process is challenging both for patients who may face barriers to informing sex partner(s) about the STI, and to services where trained staff need time to elicit sensitive information from patients in order to support PN.

Offering patients alternatives, so that they can choose the most acceptable PN method - which might differ between sex partners - is considered optimal practice.(19) For people with chlamydia, PN is most often performed through patient referral,(20,21) in which a healthcare professional advises the person with a diagnosed infection (the index patient) to inform their sex partner(s) of the need for testing and treatment and to refer them to a sexual health service (simple patient referral). This basic advice can be supplemented by information, in the form of written leaflets or website addresses, for the index patient to give to their partner(s) (enhanced patient referral). Expedited partner therapy (EPT) is a strategic approach to enhanced patient referral, widely adopted in the United States of America (USA), in which a healthcare professional gives the index patient antibiotics or a prescription for their partner(s).(22) A systematic review found that EPT results in lower proportions of index cases with repeated curable STIs than simple patient referral,(16) and a USA study demonstrated decreases in chlamydia positivity and gonorrhoea incidence at the population level.(23) However, EPT, as now widely practised in the USA, does not require a consultation with the sex partner and does not comply with UK prescribing guidance,(24) so cannot be implemented in the UK.

We therefore developed accelerated partner therapy (APT) as an adaptation of EPT to speed up the enhanced patient referral process.(25–27) APT complies with UK prescribing guidance since a healthcare professional assesses the appropriateness of the prescribed antibiotics for the partner.(28) In brief, the healthcare professional performs a telephone consultation with the sex partner in

For peer review only - http://bmjopen.bmj.com/site/about/guidelines.xhtml

private during the index patient's clinic attendance. If medically safe, the index patient receives an APT pack, containing antibiotics and self-sampling kits for STI and HIV to deliver to their sex partner(s), or the clinic may post the APT pack to the sex partner(s). In pilot studies, APT resulted in faster sex partner treatment and greater overall numbers of sex partners treated, when compared with standard PN, but lower levels of testing for HIV and other STIs, when offered without HIV testing as part of the pack.(25,26)

In the trial described here, we aimed to compare strategies for PN, specifically APT with routine PN approaches as currently practised in the UK. As part of the Limiting Undetected STi to RedUce Morbidity (LUSTRUM) programme (lustrum.org.uk), we designed a randomised controlled trial (RCT) to determine the effects of APT for people with *C. trachomatis* on biological, as well as patient-reported clinical outcomes. Specific objectives are to determine: 1. the effect of APT on the proportion of index patients who test positive for chlamydia 12-16 weeks after the PN consultation; 2. the effect of APT on the proportion of sex partners treated; 3. the cost-effectiveness of APT on long-term sexual and reproductive health outcomes, based on a linked transmission dynamic modelling study; 4. the effects of APT according to sex partner type; 5. whether APT is associated with faster treatment than standard PN, 6. the effects of APT on transmission of chlamydia at the population level, based on mathematical modelling; and to conduct: 7. a comprehensive process evaluation to understand the experiences of healthcare professionals, patients and sex partners of APT.

METHODS AND ANALYSIS

Trial design: LUSTRUM is a cross-over cluster RCT of APT offered as an additional PN method compared with standard PN alone. The APT intervention is offered at the level of the sexual health clinic, with randomisation of each clinic to either intervention or control arm in the first phase of the trial. The trial design is summarised in FIGURE 1.

FIGURE 1: Index patient and sex partner pathways during control and intervention arms

Settings: Seventeen NHS (publicly funded, free to access) specialist sexual health clinics (clusters) across England and Scotland with high volumes of positive *C. trachomatis* test results and with contrasting patient demographics. Clinics were selected from those expressing interest, based on numbers of reported chlamydia diagnoses data in the Public Health England Genitourinary Medicine Clinical Activity Dataset for STI surveillance (England) and geographical diversity (Scotland) to create three strata: London, non-London metropolitan "cities" and non-London urban "towns". A full list of study sites is included in the Acknowledgments below.

For peer review only - http://bmjopen.bmj.com/site/about/guidelines.xhtml

Eligibility criteria for index patients: Aged 16 years or older, positive test for *C. trachomatis* and/or clinical diagnosis of PID or cervicitis (women) or non-gonococcal urethritis (NGU) or epididymo-orchitis (men) and report at least one contactable sexual partner in the past six months. Index patients with PID, cervicitis, NGU and epididymo-orchitis whose test results are subsequently found to be negative for *C. trachomatis* will not be included in statistical analysis. Exclusion criteria are: groups of patients with more complex PN requirements; co-infection with other STIs including HIV, men who have had sex with men (MSM) in the past six months, and patients who have paid for or who have been paid for sex in the past six months. Patients with any clinical, social or other circumstances, such as sexual assault or insufficient English language skills to safely engage in telephone consultations, which emerge during the face-to-face consultation and make APT unsuitable will also be excluded.

Eligibility criteria for sex partners: Named as a sex partner of the index patient within the appropriate look back period (six months for chlamydia, three months for PID, one month for NGU) (29) and selected by the index patient for APT and aged 16 years or older.

Intervention: The APT intervention is a complex intervention involving index patient, healthcare professional and sex partner, situated within wider clinical care (FIGURE 1). In qualitative research studies to optimise the intervention, we explored the acceptability of individual components of the APT intervention with members of the public, sexual health clinic attenders and healthcare professionals.(30) During the intervention phase, clinics will offer APT as an additional option for eligible patients, alongside standard PN, described in BOX 1 for index patients, and BOX 2 and FIGURE 2 for sex partners. Eligible index patients who receive standard PN will receive the same follow-up as those who receive APT. If APT is no longer feasible (e.g. sex partner unavailable), standard PN will be offered instead.

For peer review only - http://bmjopen.bmj.com/site/about/guidelines.xhtml

BOX 1: Overview of APT for index patients

1. Index patient has PN consultation with healthcare professional (HCP); HCP assesses eligibility for APT.
2. Eligible index patient is offered APT alongside the clinic's other standard PN options. The patient can choose different methods for different partners.
3. Index patient telephones or messages sex partner (with or without the HCP present, according to preference) to offer immediate telephone assessment by the HCP.
4. Index patient waits in clinic while the HCP conducts APT telephone consultation in private with sex partner.
5. Index patient is informed that they will receive a follow-up telephone call in two weeks and they will either receive a chlamydia self-sampling postal kit in 12-16 weeks (preferred), or they may re-attend the clinic for testing.
6. Two-week follow-up: Research Health Adviser (RHA) telephones index patient to find out about PN outcomes with partner(s), to remind them of the repeat test, and to invite them to be contacted about taking part in a telephone interview regarding their experiences of APT (process evaluation).
7. 12 weeks: Index patient is sent a personalised text reminder about repeat test.
8. 13 weeks: Index patient is sent a self-sample kit by The Doctors Laboratory (TDL). Index patient returns self-collected sample or attends clinic for repeat testing. S/he receives results either via text message from TDL (negative results) or using routine clinic systems (positive or equivocal results). Positive results are managed according to routine clinic protocol. If the index patient does not return a self-sample or attend clinic for repeat testing, they receive a personalised text reminder eight days after the self-sample kit is sent out, followed by a telephone call from the RHA 13 days after the self-sample kit is sent out. Self-samples received >24 weeks post-PN interview are excluded.

BOX 2: Overview of APT for sex partners

1. Index patient telephones sex partner to inform them about exposure to chlamydia and offer immediate telephone assessment (APT).
2. If sex partner agrees to APT, HCP telephones them and conducts a clinical assessment in private. If appropriate, sex partner is offered an APT pack (delivered by the index patient or mailed directly). Sex partners for whom APT is inappropriate or who do not wish to continue with the APT option, will be advised by the HCP to attend clinic for further management. During the same telephone call, HCP invites sex partner to be contacted about taking part in a telephone interview regarding their experiences of APT (process evaluation).
3. Sex partner receives APT pack (FIGURE 2), which contains: antibiotics (either azithromycin or doxycycline, depending on local clinic practice); condoms; information about chlamydia, gonorrhoea, HIV and syphilis; chlamydia and gonorrhoea self-sampling kit, HIV and syphilis self-sampling kit, and information leaflet about how to take a sample (including link to an explanatory online video: lustrum.org.uk/test-and-treat); request form for the sample to be processed by the lab; envelope for return of self-sampling kits; and APT pack packaging (envelope or small box, no branding or other identifiable markings, and which fits through standard letterbox).
5. Sex partner completes self-sampling, labels, and returns samples for testing.
6. Sex partner takes antibiotic treatment.
7. Sex partner informed of test results by text (negative results) or routine clinic processes; positive results are managed according to routine care.

For peer review only - http://bmjopen.bmj.com/site/about/guidelines.xhtml

FIGURE 2: Contents of sex partner APT pack

Control: During the control phase, each clinic follows their usual protocols for standard PN. All clinics conduct enhanced patient referral, including verbal information about notifying partners plus additional information (written leaflets or signposting to a website). The healthcare professional will occasionally notify partners on behalf of the patient, if requested. Follow-up telephone calls and repeat testing will be the same as those during the APT phase.

Outcomes: The primary clinical outcome is the proportion of index patients with a positive test result for *C. trachomatis* 12-16 weeks after the PN consultation. A repeated positive test result after treatment is a proxy for re-infection from an untreated partner, assuming that other reasons for a repeat positive test result are distributed equally between intervention and control arms. This outcome has been used in RCTs of PN interventions seeking to demonstrate prevention of transmission at the level of the individual.(16)

Secondary clinical outcomes are: 1) the proportion of sex partners treated two weeks after the initial PN consultation; 2) numbers of partners treated per index patient; 3) time to partner treatment; and 4) number of patients notified per index patient. These outcomes will be ascertained by index patient reports at the two-week follow-up telephone call with the RHA. Additional secondary outcomes include exploring the acceptability of the APT intervention to index patients and sex partners through qualitative telephone interviews with patients and partners as part of the process evaluation. We will determine rates of STI and HIV testing in sex partners and proportions of positive test results for STIs and HIV among sex partners who return postal self-sampling kits contained in APT packs. We will determine costs associated with the intervention in economic evaluation studies based on costings of the trial. We will record any adverse events using local activity logs at each trial site and report them to the trial sponsor and ethics committee.

Sample size: The trial is designed to determine superiority of APT as an adjunct to standard PN compared with standard PN. Our calculation is based on recruiting an average of 160 index patients per clinic per trial phase from the 17 participating clinical services (total 5440 patients) and a coefficient of variation in the number recruited of 0.5. We expect that 50% of recruited patients (80 per clinic per phase and 2720 total) will contribute to the analysis of the primary outcome because we restrict this analysis to patients with *C. trachomatis* detected at baseline, we assume that 60% of index patients will provide a repeat sample for *C. trachomatis* detection and we expect that 10 to 25% of patients in the control arm will have *C. trachomatis* detected at the 12-16 weeks follow up.(16,31,32) This sample size provides 80% power (at the 5% significance level) to detect a fall in *C. trachomatis* positivity from 10% to 5%, and 82% power to detect a fall from 25 to 17%. We consider

For peer review only - http://bmjopen.bmj.com/site/about/guidelines.xhtml

such reductions would be reasonable if around half the index patients in the intervention phase select APT for some or all their partners, but our calculation does not assume any specific value of APT uptake. A period of six months in the control condition and six months in the intervention condition should be sufficient to reach this target. Related to our key secondary outcome (the proportion of sex partners treated), for simplicity, we consider power to detect an effect on the proportion of index patients with one or more partner treated. This sample size provides 87% power to detect an increase from 60% in the control arm to 70% in the intervention arm.(17)

Sample size calculations are guided by Giraudeau et al. (33) and performed as if the trial were a standard cluster RCT with 17 clinics in each arm. The cross-over design in our trial, with each cluster contributing data to both intervention and control conditions, gives more information about the intervention effect and so greater power than stated above. Our calculations assume an intra-cluster correlation (ICC) of 0.02, which is not based on published data. We believe that our power calculations are likely to be conservative because any loss from a higher ICC will be more than offset by the gain from the cross-over design.

Recruitment: During the initial PN consultation with the index patient, the healthcare professional will assess eligibility for the study for all patients with a laboratory positive test result for chlamydia or a clinical diagnosis of NGU/epididymo-orchitis (men) or PID/ cervicitis (women). During the intervention phase, the healthcare professional will offer eligible patients APT in addition to standard methods of PN. The trial data manager will monitor recruitment and patient uptake of the APT intervention throughout the trial period supplying monthly recruitment figures to the Trial Management Group (TMG) and Data Monitoring Committee (DMC).

Randomisation / allocation sequence: The random allocation of clinics, to intervention or control arm first, was conducted through random permutation within strata, using computer-generated random numbers in Stata 15 software. In four strata, the two clinics were part of one NHS trust and were considered as pairs. One stratum contained five clinics from large cities and another contained three clinics from smaller settlements. Given the odd numbers of clinics in these strata, we used random assignment to determine whether two or three clinics would implement the intervention first in the larger stratum (correspondingly two or one in the smaller stratum to ensure balance). One further clinic, the last (17th) to start recruitment was allocated through simple randomisation, giving a total of nine clinics allocated to implement the intervention first and eight to first follow routine practice. To remove the potential for allocation bias arising because allocation codes were generated after clinics had already been recruited to the trial, one statistician generated the

For peer review only - http://bmjopen.bmj.com/site/about/guidelines.xhtml

allocation codes and another randomly permuted the clinic names within the strata. Only then, a third person matched the two to reveal the allocations and inform the clinic manager.

Blinding: Blinding of allocation will not be possible for healthcare professionals, patients or research staff. A statistician blinded to allocation will replicate the analysis of the primary outcome.

Procedure for collecting data: Healthcare professionals will record their consultations in real time and download data needed for clinical purposes into the patient's clinic record. They will use RELAY, a bespoke data collection tool. RELAY is a secure web-based data collection platform, which incorporates different interfaces allowing specified levels of access to healthcare professionals, research health advisers, data manager, trial manager and the research team. It is hosted on secure servers and compliant with NHS data storage requirements. We developed RELAY for the LUSTRUM trial, based on previous versions of data collection tools in our pilot studies of APT.(25,26)

Statistical analysis: Analysis will be by intention-to-treat. For the primary, and other quantitative outcomes, we will fit random effects models with fixed effects for clinic and intervention condition, together with a random effect to acknowledge the clustering of index patients for each combination of clinic and for each period.(34) The primary and key secondary outcomes are binary, so we will use logistic regression models. The intervention effect will be expressed as an odds ratio with 95% confidence interval. Models for secondary outcomes with outcomes quantified for each sexual contact will include additional random effects for the index patient.

We anticipate loss to follow-up of around 40% of participants, raising potential concerns over bias. Before conducting our analysis of the primary outcome we will analyse predictors of follow-up from among a pre-specified list of characteristics of the index patients that will be recorded at baseline and which are considered to be likely predictors of the primary outcome. In the primary analysis of the primary outcome we will include these as covariates in our regression model along with any other key predictors of the outcome. This approach should reduce bias from loss-to-follow-up but we will also conduct further sensitivity analyses based on multiple imputation in which we will allow those lost to-follow-up to be more, and then less, likely to be chlamydia positive at three months than those not lost to follow-up with the same baseline covariates. These sensitivity analyses will follow the approach of Carpenter et al.,(35) to assess the robustness of our conclusions to different pre-specified assumptions about missing data.

Exclusions from analyses: The intervention is implemented at the clinic level and applied to all eligible patients with service-level, rather than individual, consent. Patients can opt-out of their data being used for research (see supplementary file 1) Once a clinic has begun the allocated intervention

For peer review only - http://bmjopen.bmj.com/site/about/guidelines.xhtml

then all eligible patients added to the RELAY web tool will be included in analysis, following the spirit of an intention-to-treat analysis and the all-randomised population. However, in the unlikely event of a substantial period in which any clinic is subsequently unable to implement the intervention for any reason then we will also conduct an analysis that only includes patients attending the clinic while it is able to offer the intervention, in the spirit of a per-protocol analysis and a protocol compliant population. The inclusion of patients in analysis will not be influenced by whether the patient does or does not take up the offer of APT.

Patients who present with conditions managed at first attendance as presumptive urogenital chlamydia but who subsequently are found to have negative chlamydia tests will be excluded from analyses.

Trial timeline: There will be a four-month run in period (July – October 2018), consisting of rolling clinic set up including training for healthcare professionals and a period of at least two weeks of baseline data collection when healthcare professionals will use RELAY to record standard PN data. Research staff will monitor completeness of baseline data collection. The data will be used at the end of the trial to help interpret the trial findings. Then half of the clinics will enter intervention phase while the other half enter control phase, according to the randomisation schedule. The trial is planned to start 22nd October 2018.

At the end of the first six-month trial phase (October 2018 – April 2019) there will be a two-week washout period where clinics will not offer APT to patients and follow their standard PN practice procedures. Clinics which start with the control phase will also observe the two-week wash-out to align the trial time periods. Then clinics cross over to the opposite arm (intervention or control) for phase two (for six months, May – November 2019); patient recruitment is planned to end 17th November 2019. The total duration of the trial will be 19 months, allowing for a three-month follow-up period to enable outcome data collection to be completed for all patients in the second trial phase. Clinics which do not start phase one of the trial in November 2018 will complete recruitment in November 2019 and trial phases will be condensed. The LUSTRUM programme as a whole runs from April 2016 to March 2021.

Process evaluation: We will conduct an integrated process evaluation, which involves a suite of sub-studies with index patients, sex partners and healthcare professionals. In brief these studies include: 1) qualitative telephone interviews with a sample of index patients who accepted APT for one or more sex partner, which will take place up to eight weeks after their first PN consultation; 2) qualitative telephone interviews with a sample of sex partners who received APT, up to eight weeks after their APT consultation; 3) qualitative telephone interviews with healthcare professionals who

For peer review only - http://bmjopen.bmj.com/site/about/guidelines.xhtml

delivered APT, which take place up to 16 weeks after the clinic offers APT during the intervention phase; 4) focus groups with healthcare professionals to explore the context, extent and fidelity of the intervention delivery, which will take place up to 16 weeks after the clinic offers APT during the intervention phase.

All potential participants will receive the relevant participant information sheet and the researcher will discuss this information with participants before seeking informed consent to participate (see supplementary files 2-5). All telephone interviews will be audio-recorded with informed verbal consent prior to participation, transcribed verbatim and analysed using interpretative phenomenological analysis (36) to understand lived experiences of using APT. The expected duration of telephone interviews is 30-60 minutes. Focus groups will be audio-recorded with informed written consent prior to participation, transcribed verbatim and data will be analysed using the Framework approach.(37) Data collected will assess intervention fidelity, healthcare professionals' APT training (the behaviour change techniques they received), and their experience of APT delivery (the behaviour change techniques they themselves delivered) in relation to a range of feasibility issues such as impact upon clinic flow and wider systemic and cultural issues.

Health economics evaluation: The aim of the economic evaluation is to determine the relative cost effectiveness of APT compared with standard PN. If APT reduces the number of re-infected cases, there will be cost implications for the healthcare sector and for society as a whole because of effects on the incidence of complications. Cost effectiveness will be determined in two ways using two separate economic analyses which will explore the costs and consequences of the alternative interventions and report results in terms of Incremental Cost Effectiveness Ratios (ICERs) if applicable. The two analyses are: (i) within trial economic evaluation which will report the terms of natural units including *cost per case of re-infection avoided* (ii) model-based economic evaluation which will report results in terms of cost per QALY based on new data from recent research.(38)

The within trial evaluation will use only data collected within the trial and will draw upon data collected through provision of APT up until four months post-intervention when the index cases are re-tested. The evaluation will adopt the perspective of the NHS and will be based on an outcome of cost per case of re-infection avoided. It is acknowledged that this is an intermediate outcome. We will build on the experience of exploring the cost effectiveness of alternative versions of APT in previous exploratory and pilot studies.(25,26)

The trial will not capture the effects of APT on the transmission of chlamydia, or on female reproductive tract complications, such as PID. As for previous economic evaluations of chlamydia screening interventions,(39) we will develop a dynamic model of the epidemiology of *C. trachomatis*

For peer review only - http://bmjopen.bmj.com/site/about/guidelines.xhtml

transmission, the APT intervention and its outcomes, which will use data from the trial. The model based economic evaluation and deterministic transmission dynamic model upon which the economic evaluation will be based, will be reported in detail in a separate publication.

Trial monitoring: The Trial Management Group (TMG) is responsible for the day-to-day design, delivery, and management of the trial.

The Trial Steering Committee (TSC) is an executive and independent body, providing overall supervision of the trial and ensuring that it is being conducted in accordance with the principles of Good Clinical Practice and the relevant regulations. The TSC will approve the trial protocol and any protocol amendments and provide advice to investigators on all aspects of the trial. The TSC will monitor trial progress including recruitment, data completeness, and losses to follow-up and ensure that there are no major and/or unexplained deviations from the trial protocol.

The Data Monitoring Committee (DMC) will be responsible for reviewing interim trial data and reporting back to the sponsor on the future management of the trial. The DMC will use data accumulated throughout the trial to inform guidance and recommendations for the continued ethical conduct of the trial. The APT intervention is a short, one-off intervention provided to patients as an additional PN option which complements existing standard care practices. As such, the risk of APT to patients is minimal. We will not conduct a formal interim analysis.

We will collect reports of adverse events relating to either the intervention or participation in the trial, which will be reviewed by the TSC and reported to the sponsor and ethics committee as appropriate. The TSC could recommend the trial be stopped if members are sufficiently concerned about these events. The trial will not be stopped early due to demonstration of efficacy, harm or futility in the primary outcome.

Patient and public involvement: The LUSTRUM PPI Group consists of 27 lay people, with a broad mix of demographic characteristics and a range of sexual health experiences. The group were first involved in the design of the research at the grant application stage. Their opinions guided the trial protocol and topic guides for the process evaluation interviews. The trial is conducted by qualified healthcare professionals and, as such, it is not feasible for the group to undertake any aspects of the data collection. The group strongly supported the service-level consent design which reduces the burden of participating in the research. Lay summaries of our findings will be circulated to the group to gain their perspective and feedback on our results; this may inform further analytical decisions and inputs.

ETHICS and DISSEMINATION

For peer review only - http://bmjopen.bmj.com/site/about/guidelines.xhtml

As this is a pragmatic cluster randomised trial, we will seek consent for trial participation from lead clinicians at participating clinics (service-level) and will not seek individual informed consent from index patients other than for the process evaluation studies. Following Weijer et al.,(40) we believe that APT is a complex, “low-risk” healthcare delivery intervention. APT is offered in addition to standard PN and operationalised as a supplement to usual care, thus index patients have the choice of taking up APT or not. It is widely accepted that individual consent may not be essential in such trials,(41) in which the situation is analogous to the introduction of changed processes in routine services.(31) Notably, individual level consent is thought to have contributed to low recruitment numbers in a previous study of APT.(26) Individual informed consent will be sought from all participants in the process evaluation.

Study endpoints, whether negative or positive, will be reported and disseminated through the following channels: 1. Research findings will be published in journals with open access within 6 months of publication. 2. Research findings will be presented at UK and international meetings orally or via posters. 3. A report of the findings (in the form of the funder’s final report) will be freely available on both the funder’s website and the LUSTRUM website after publication in scientific journals. A link to the report will be circulated to stakeholders, collaborators and participating organisations, together with lay summaries of the study on the LUSTRUM website. 4. We will make presentations to community groups and clinic user groups as appropriate. 5. We will make continued use of social media channels to report findings after publication in scientific journals, in order to increase the “reach” of our findings to the public. 6. The anonymised participant level dataset, and statistical code for generating the results will be archived in a data repository after publication of the final report.

For peer review only

For peer review only - http://bmjopen.bmj.com/site/about/guidelines.xhtml

REFERENCES

1. UNAIDS. Sexually transmitted diseases: policies and principles for prevention and care. Geneva; 1999. Available from: https://www.unaids.org/en/resources/documents/1999/19990519_una97-6_en.pdf
2. Althaus CL, Turner KM, Mercer CH, Auguste P, Roberts TE, Bell G, et al. Effectiveness and cost-effectiveness of traditional and new partner notification technologies for curable sexually transmitted infections: observational study, systematic reviews and mathematical modelling. Health Technol Assess (Rockv). 2014 Jan;18(2):1–100, vii–viii.
3. Public Health England. Table 1: STI diagnoses and rates in England by gender, 2009 to 2018. London; 2019. Available from: https://www.gov.uk/government/statistics/sexually-transmitted-infections-stis-annual-data-tables
4. Health Protection Scotland. Surveillance report Genital chlamydia and gonorrhoea infection in Scotland: laboratory diagnoses 2009-2018. Health Protection Scotland; 2019. Available from: https://www.hps.scot.nhs.uk/web-resources-container/genital-chlamydia-and-gonorrhoea-infection-in-scotland-laboratory-diagnoses-2009-2018
5. Public Health Wales. HIV and STI trends in Wales - Surveillance Report [Internet]. Public Health Wales; 2018. Available from: http://www.wales.nhs.uk/sitesplus/documents/888/HIV and STI trends in Wales Report 2018_2017 -Surveillance report.pdf
6. Holmes KK, Sparling FP, Stamm WE, Piot P, Wasserheit JN, Corey L, et al., editors. Sexually Transmitted Diseases. Fourth Edi. New York, London: McGraw-Hill Medical; 2008.
7. Hosenfeld CB, Workowski KA, Berman S, Zaidi A, Dyson J, Mosure D, et al. Repeat infection with chlamydia and gonorrhea among females: A systematic review of the literature. Sex Transm Dis. 2009 Aug;36(8):478–89.
8. Scott Lamontagne D, Baster K, Emmett L, Nichols T, Randall S, McLean L, et al. Incidence and reinfection rates of genital chlamydial infection among women aged 16-24 years attending general practice, family planning and genitourinary medicine clinics in England: a prospective cohort study by the Chlamydia Recall Study Advisory Group. Sex Transm Infect. 2007 Jul;83(4):292–303.
9. Walker J, Tabrizi SN, Fairley CK, Chen MY, Bradshaw CS, Twin J, et al. Chlamydia trachomatis incidence and re-infection among young women - behavioural and microbiological characteristics. PLoS One. 2012 May 25;7(5).
10. Public Health England. Sexually Transmitted Infections and Chlamydia screening in England: 2018. London, England; 2019. Available from: https://assets.publishing.service.gov.uk/government/uploads/system/uploads/attachment_data/file/806118/hpr1919_stis-ncsp_ann18.pdf
11. Health Protection Scotland. Surveillance report Genital chlamydia and gonorrhoea infection in Scotland: laboratory diagnoses 2009-2018. Health Protection Scotland; 2019.
12. Public Health Wales. HIV and STI trends in Wales - Surveillance Report. Public Health Wales; 2018.
13. Public Health England. Table 2 (a - 1): Number of chlamydia diagnoses in England by gender, sexual risk & age group, 2014 - 2018. London, England; 2019. Available from: https://www.gov.uk/government/statistics/sexually-transmitted-infections-stis-annual-data-tables
14. Department of Health. Improving outcomes and supporting transparency Part 2: summary technical specifications of public health indicators. In: Public Health Outcomes Framework 2016 to 2019. 2017.
15. Heijne JCM, Herzog SA, Althaus CL, Tao G, Kent CK, Low N. Insights into the timing of repeated testing after treatment for Chlamydia trachomatis: data and modelling study. Sex Transm Infect. 2013 Feb;89(1):57–62.

For peer review only - http://bmjopen.bmj.com/site/about/guidelines.xhtml

16. Ferreira A, Young T, Mathews C, Zunza M, Low N. Strategies for partner notification for sexually transmitted infections, including HIV. In: Low N, editor. Cochrane Database of Systematic Reviews [Internet]. Chichester, UK: John Wiley & Sons, Ltd; 2013.
17. McClean H, Carne CA, Sullivan AK, Radcliffe KW, Ahmed-Jushuf I, National Audit Group of British Association for Sexual Health and HIV. Chlamydial partner notification in the British Association for Sexual Health and HIV (BASHH) 2011 UK national audit against the BASHH Medical Foundation for AIDS and Sexual Health Sexually Transmitted Infections Management Standards. Int J STD AIDS. 2012 Oct;23(10):748–52.
18. Healthcare Improvement Scotland. Improving Sexual Health Services in Scotland: Integration and Innovation National Overview. 2011.
19. Trelle S, Shang A, Nartey L, Cassell J a, Low N. Improved effectiveness of partner notification for patients with sexually transmitted infections: systematic review. BMJ. 2007;334(7589):354.
20. Fontaine J, Sfetcu O, Van De Laar M, Martin-Hilber A, Röllin A, Redmond S, et al. Public health benefits of partner notification for sexually transmitted infections and HIV.
21. Centers for Disease Control and Prevention. Chlamydia Infections Guidelines. In: 2015 STD Treatment Guidelines. Atlanta; 2015.
22. Centre for Disease Control and Prevention. Expedited partner therapy in the management of sexually transmitted diseases. Atlanta; 2006.
23. Golden MR, Kerani RP, Stenger M, Hughes JP, Aubin M, Malinski C, et al. Uptake and population-level impact of expedited partner therapy (EPT) on Chlamydia trachomatis and Neisseria gonorrhoeae: the Washington State community-level randomized trial of EPT. PLoS Med. 2015 Jan;12(1):e1001777.
24. General Medical Council. Good medical practice. London, England: GMC; 2013.
25. Estcourt C, Sutcliffe L, Cassell J, Mercer CH, Copas A, James L, et al. Can we improve partner notification rates through expedited partner therapy in the UK? Findings from an exploratory trial of Accelerated Partner Therapy (APT). Sex Transm Infect. 2012 Feb;88(1):21–6.
26. Estcourt CS, Sutcliffe LJ, Copas A, Mercer CH, Roberts TE, Jackson LJ, et al. Developing and testing accelerated partner therapy for partner notification for people with genital Chlamydia trachomatis diagnosed in primary care: A pilot randomised controlled trial. Sex Transm Infect. 2015 Dec;91(8):548–54.
27. Sutcliffe L, Brook MG, Chapman JL, Cassell JM, Estcourt CS. Is accelerated partner therapy a feasible and acceptable strategy for rapid partner notification in the UK?: A qualitative study of genitourinary medicine clinic attenders. Int J STD AIDS. 2009 Sep;20(9):603–6.
28. General Medical Council. Good practice in prescribing and managing medicines and devices. London, England: GMC; 2013.
29. Mcclean H, Radcliffe K, Sullivan A, Ahmed-Jushuf I. 2012 BASHH Statement on Partner Notification for Sexually Transmissible Infections. Int J STD AIDS. 2013;24(4).
30. Flowers P, Pothoulaki M, Woode Owusu M, Vojt G, Mapp F, Mercer CH, et al. P079 Using theory & evidence to optimise an Accelerated Partner Therapy intervention in the context of a chlamydia partner notification trial. STI HIV 2019 World Congr Vancouver. 2019;95(Suppl 1).
31. Cassell J, Young A. Why we should not seek individual informed consent for participation in health services research. J Med Ethics. 2002 Oct 1;28(5):313–7.
32. Van Der Helm JJ, Koekenbier RH, Van Rooijen MS, Schim Van Der Loeff MF, De Vries HJC. What Is the Optimal Time to Retest Patients with a Urogenital Chlamydia Infection? A Randomized Controlled Trial. Sex Transm Dis. 2018 Feb 1;45(2):132–7.
33. Giraudeau B, Ravaud P, Donner A. Sample size calculation for cluster randomized cross-over trials. Stat Med. 2008 Nov 29;27(27):5578–85.
34. Turner RM, White IR, Croudace T, PIP Study Group. Analysis of cluster randomized cross-over trial data: a comparison of methods. Stat Med. 2007 Jan 30;26(2):274–89.
35. Carpenter JR, Kenward MG, White IR. Sensitivity analysis after multiple imputation under

For peer review only - http://bmjopen.bmj.com/site/about/guidelines.xhtml

missing at random: a weighting approach. Stat Methods Med Res. 2007 Jun 2;16(3):259–75.
36. Smith JA, Flowers P, Larkin M. Interpretative phenomenological Analysis. London, Thousand Oaks, New Delhi, Singapore: Sage Publications Ltd.; 2009.
37. Ritchie J, Lewis J. Qualitative Research Practice. London: SAGE Publications; 2003.
38. Okeke Ogwulu C. Developing and piloting approaches for the valuation of outcomes associated with sexually transmitted infections [Internet]. University of Birmingham; 2018. Available from: https://ethos.bl.uk/OrderDetails.do?uin=uk.bl.ethos.760507
39. Low N, McCarthy A, Macleod J, Salisbury C, Campbell R, Roberts TE, et al. Epidemiological, social, diagnostic and economic evaluation of population screening for genital chlamydial infection. Health Technol Assess. 2007 Mar;11(8):iii–iv, ix–xii, 1-165.
40. Weijer C, Grimshaw JM, Taljaard M, Binik A, Boruch R, Brehaut JC, et al. Ethical issues posed by cluster randomized trials in health research. Trials. 2011 Apr 20;12:100.
41. Campbell MK, Weijer C, Goldstein CE, Edwards SJL. Do doctors have a duty to take part in pragmatic randomised trials? BMJ. 2017.

Required statements:

Trial Protocol Version 5.0 [29-07-19]

Acknowledgements: The authors would like to thank the following**:**

- **Trial Steering Committee:** Simon Barton (chair), Robbie Currie, David Crundwell, Artemis Koukounari, Lynis Lewis, Alec Miners, Emmanuel Rollings-Kamara, Rachel Shaw, Emma Thompson
- **Data Monitoring Committee:** Simon Barton, David Crundwell, Rebecca Turner, Artemis Koukounari
- **All members of the LUSTRUM Patient and Public Involvement Group**
- **Participating Centres:** Barking, Havering and Redbridge University Hospitals NHS Trust (A. Umaipalan), Barts Health NHS Trust (V. Apea), Buckinghamshire Healthcare NHS Trust (J. Sherrard), Chelsea and Westminster Hospital NHS Foundation Trust (C. Evans), Croydon Health Services NHS Trust (D. Phillips), Manchester University NHS Foundation Trust (G. Schembri), Midlands Partnership NHS Foundation Trust (J. Dhar), NHS Greater Glasgow and Clyde (R. Nandwani), Northamptonshire Healthcare NHS Foundation Trust (S. Herbert), Royal Berkshire NHS Foundation Trust (F. Chen), Royal Bournemouth and Christchurch Hospitals NHS Foundation Trust (K. Schroeder), Solent NHS Trust (R. Patel), University Hospitals Birmingham NHS Foundation Trust (J. Ross)

The authors would also like to thank epiGenesys who developed the RELAY software; Soazig Clifton (support with ethics submission); Jo Gibbs (support on RELAY development); Kay Musonda (administrative support); and the staff, management and patients involved in the study at all the participating sexual health clinics.

Authors' contributions: CSE is Chief Investigator of the study. CSE, JC, NL, TR, AnC, AJ, CHM, JS, MS, PF, RN and SW conceived the study and secured funding. They are responsible for the planning and delivery of the study. PF leads the process evaluation; TR leads on the health economics evaluation; and NL leads on the mathematical modelling. AnC is the trial statistician.

ARH, FM, MWO are responsible for coordination of the study. ARH, FM, MWO, KP, COO, SB, AlC, TM, MP, GV and AT are responsible for operationalisation of the study procedures. FM, MWO, KP, COO, MP and GV will contribute to data collection and analysis. ARH, SB and AlC will contribute to data

For peer review only - http://bmjopen.bmj.com/site/about/guidelines.xhtml

collection. CA and NL are responsible for the mathematical modelling. AT is responsible for trial data management and will contribute to data analysis.

All authors contributed to the development of the study design and establishment of procedures.

CSE led on preparing the manuscript. All authors critically reviewed and approved the final version.

Funding statement: This work presents independent research funded by the National Institute for Health Research (NIHR) under its Programme Grants for applied research Programme (Reference Number RP-PG-0614-20009). The views expressed are those of the author(s) and not necessarily those of the NHS, the NIHR or the Department of Health. The funders had no role in study design, collection, management, analysis, and interpretation of data; writing of the report; and the decision to submit the report for publication.

Trial Sponsor: Noclor, Central and North West London NHS Trust. The sponsor had no role in study design, collection, management, analysis, and interpretation of data; writing of the report; and the decision to submit the report for publication.

Competing interests statement: The authors have no competing interests.

Data sharing statement, Anonymised participant level dataset, and statistical code for generating the results will be archived in a data repository after publication of the final report.

Patient consent statement: Not required

Ethical approval: Ethical approval from London - Chelsea Research Ethics Committee (18/LO/0773).

For peer review only

For peer review only - http://bmjopen.bmj.com/site/about/guidelines.xhtml

FIGURE 1: Index patient and sex partner pathways during control and intervention arms

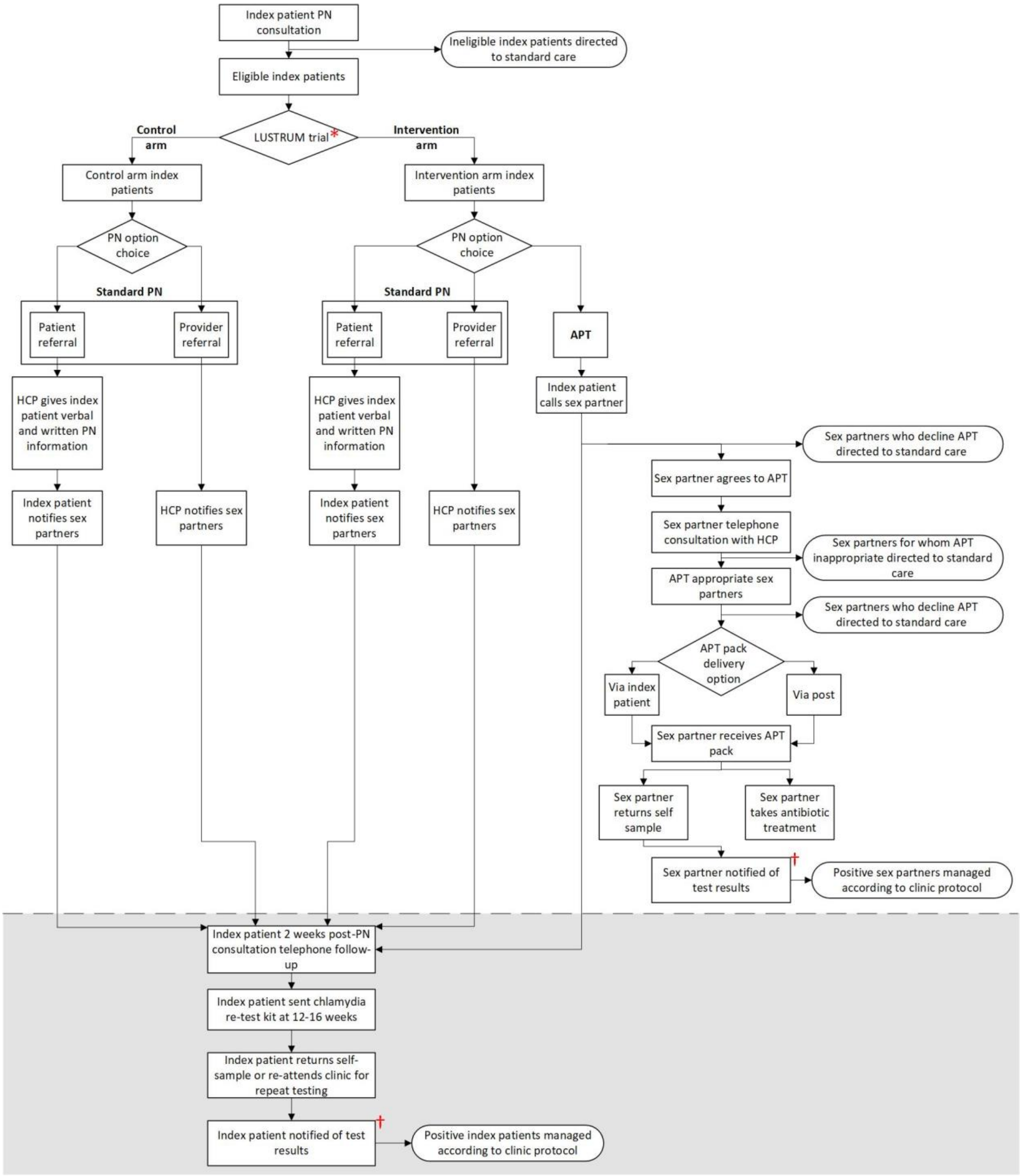

PN, Partner Notification; APT, Accelerated Partner Therapy; HCP, Healthcare Professional

* Random allocation to intervention or control arm in the first phase of the trial

† Index patients and sex partners are notified of results via two pathways: 1. Negative results: individual receives a text from The Doctors Laboratory (TDL); 2. Positive / equivocal results: individual receives results directly from clinic

FIGURE 2: Contents of sex partner APT pack

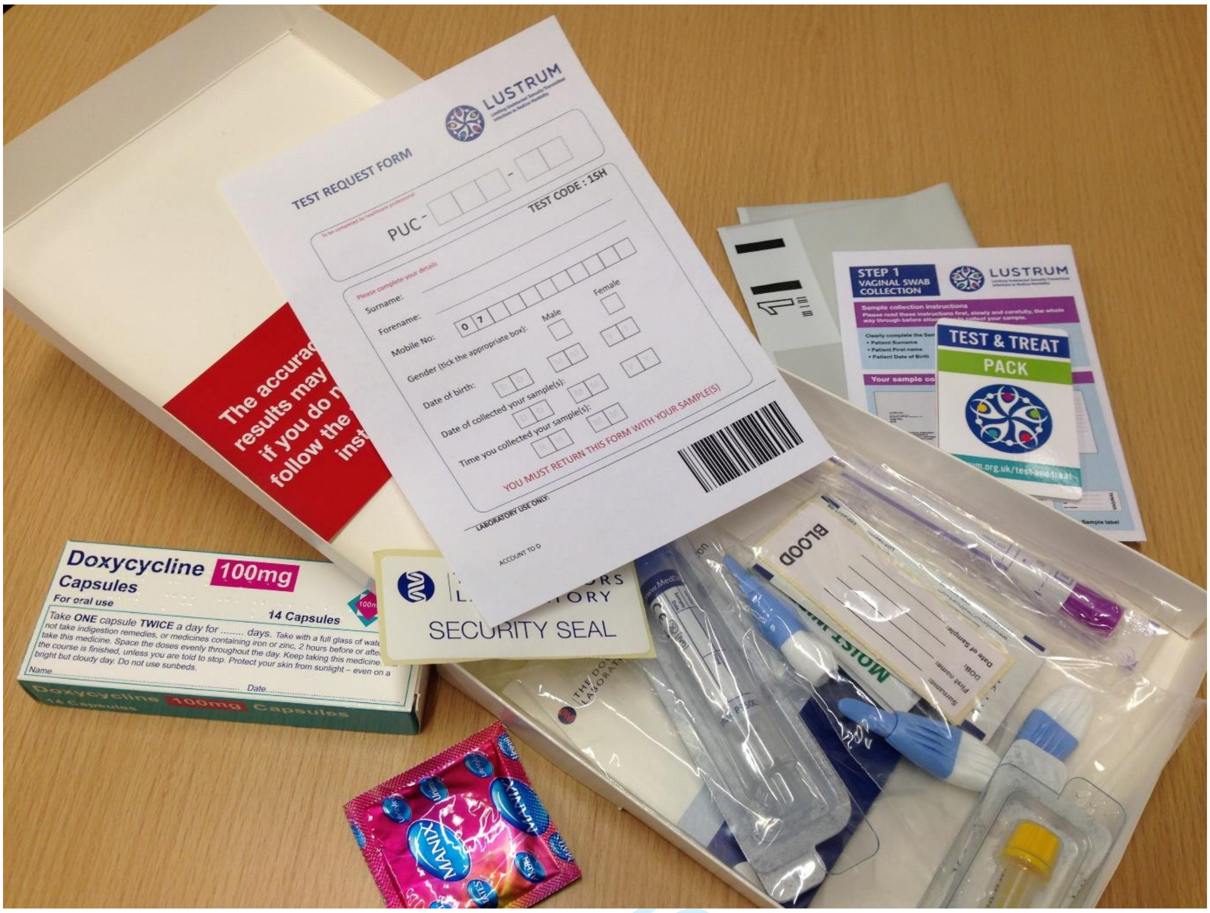

Pack contains: Antibiotics; Condoms; LUSTRUM TEST & TREAT leaflet; Vulvo-vaginal swab kit OR urine sampling kit; Blood sampling kit; Instruction leaflet for sampling kits; Test request form for sample processing; Pre-paid return post envelope; Security seal sticker; Attention card

[Trust logo]

Your Data Matters to the NHS

Information about your health and care helps us to improve your individual care, speed up diagnosis, plan your local services and research new treatments.

In May 2018, the strict rules about how this data can and cannot be used were strengthened. The NHS is committed to keeping patient information safe and always being clear about how it is used.

You can choose whether your patient information is used for research at [name of clinic].

Version 1.0 [19-07-18]

For peer review only

You can choose whether your patient information is used for research at [name of clinic].

How your data is used

Your health and care information is used to improve your individual care. It is also used to help us research new treatments and plan your local services. We only use *anonymised* data for research studies – this is information which does not identify you.

Who can use your patient information for research and planning?

It is used by the NHS, local authorities, university and hospital researchers, medical colleges and pharmaceutical companies researching new treatments.

Making your data opt-out choice

You can choose to opt out of sharing your anonymised patient information for research at [name of clinic]. There may still be times when your patient information is used: for example, during an epidemic where there might be a risk to your or to other people's health. You can also still consent to take part in a specific research project.

Will choosing this opt-out affect your care and treatment?

No, your patient information will still be used for your individual care. Choosing to opt out will not affect your care and treatment.

What should I do next?

You do not need to do anything if you are happy about how your patient information is used at [name of clinic].

If you do not want your anonymised patient information to be used for research at [name of clinic], you can choose to opt out. Please tell our reception staff.

You can change your choice at any time.

To find out more, please contact: [name of clinical lead at clinic]

For peer review only

[INSERT SITE LOGO]

Interview consent form (Patients & Sex Partners) - Process Evaluation Studies 1&2
Version 2.1 [22-06-18]
IRAS 245000

Appendix E

PE Studies 1 & 2

Title of the project: Accelerated Partner Therapy (APT) Process Evaluation Study
Centre Number:
Study Number:

VERBAL CONSENT FORM
(Sexual Health clinic patients and sex partners)

Researcher to initial boxes

Name of Chief Investigator: Prof. Claudia Estcourt

1. I confirm that I have read and understood the information sheet dated 22-06-18, version 2.1 for the above study. I have had the opportunity to consider the information, ask questions and I am satisfied with the answers. ☐

2. I am aged between 16 and 65 years of age. ☐

3. I understand that my participation is voluntary and that I am free to withdraw from the study at any time without giving a reason, without my medical care or legal rights being affected. ☐

4. I understand that relevant sections of my demographic information and data collected during the LUSTRUM Accelerated Partner Therapy (APT) Chlamydia Partner Notification Trial may be looked at by researchers from Glasgow Caledonian University and University College London, where it is relevant to my taking part in this study. I give permission for these individuals to have access to my records. ☐

5. I understand that the information collected about me will be used to support other research in the future, and may be shared anonymously with other researchers. ☐

6. I understand that if I or someone else is believed to be in danger or at risk of significant harm, this will be reported directly to [name], [Lead for safeguarding] and Professor Claudia Estcourt, Chief Investigator on the study. Any allegations of poor practice discovered during the study will be reported directly to [name], Head of Service for GU/HIV medicine and [name], Service Manager. ☐

7. I give permission for the interview to be audio-recorded and transcribed. ☐

8. I agree to the use of direct quotations from my interview in publications, reports and/or presentations provided that anonymity is preserved. I understand that the results of this study may be published; however my anonymity will be preserved. ☐

9. I agree to take part in the above study. ☐

10. I confirm that I am still happy for my data to be included in the study (**to be completed at the end of the session**). ☐

____________________	____________________	____________________
Name of participant	Date	Time verbal consent obtained
____________________	____________________	____________________
Name of person taking consent	Date	Signature

Central and North West London NHS
NHS Foundation Trust

LUSTRUM
Limiting Undetected Sexually Transmitted Infections to RedUce Morbidity

For peer review only

[INSERT SITE LOGO]

When completed: 1 for researcher site file.

For peer review only

Central and North West London NHS
NHS Foundation Trust

LUSTRUM
Limiting Undetected Sexually Transmitted Infections to RedUce Morbidity

[INSERT SITE LOGO]

Interview consent form (Healthcare Professionals)
Process Evaluation Study 3
Version 2.1 [22-06-18]
IRAS 000245

Appendix E **PE Study 3**

Title of the project: Accelerated Partner Therapy (APT) Process Evaluation Study

Centre Number:

Study Number:

For peer review only

VERBAL CONSENT FORM

(Healthcare Professionals – Telephone Interviews)

Name of Chief Investigator: Prof. Claudia Estcourt

Researcher to initial boxes

1. I confirm that I have read and understood the information sheet dated 22-06-18 version 2.1 for the above study. I have had the opportunity to consider the information, ask questions and I am satisfied with the answers. ☐
2. I understand that my participation is voluntary and that I am free to withdraw from the study at any time without giving a reason. ☐
3. I understand that the information collected about me will be used to support other research in the future, and may be shared anonymously with other researchers. ☐
4. I understand that if I or someone else is believed to be in danger or at risk of significant harm, this will be reported directly to [name], [Lead for safeguarding] and Professor Claudia Estcourt, Chief Investigator on the study. Any allegations of poor practice discovered during the study will be reported directly to [name], Head of Service for GU/HIV medicine and [name], Service Manager. ☐
5. I give permission for the interview to be audio-recorded and transcribed ☐
6. I agree to the use of direct quotations from my interview in publications, reports and/or presentations provided that anonymity is preserved. I understand that the results of this study may be published; however my anonymity will be preserved. ☐
7. I agree to take part in the above study. ☐
8. I confirm that I am still happy for my data to be included in the study (**to be completed at the end of the session**). ☐

Central and North West London NHS
NHS Foundation Trust

LUSTRUM
Limiting Undetected Sexually Transmitted Infections to RedUce Morbidity

[INSERT SITE LOGO]

Interview consent form (Healthcare Professionals)
Process Evaluation Study 3
Version 2.1 [22-06-18]
IRAS 000245

Name of participant	Date	Time verbal consent obtained
Name of person taking consent	Date	Signature

When completed: 1 for researcher site file.

Central and North West London NHS
NHS Foundation Trust

LUSTRUM
Limiting Undetected Sexually Transmitted Infections to RedUce Morbidity

For peer review only

[INSERT SITE LOGO]

Interview consent form (Healthcare Professionals)
Process Evaluation Study 3
Version 2.1 [22-06-18]
IRAS 000245

Appendix E

PE Study 3

Title of the project: Accelerated Partner Therapy (APT) Process Evaluation Study

Centre Number:

Study Number:

CONSENT FORM

(Healthcare Professionals – Interviews)

Name of Chief Investigator: Prof. Claudia Estcourt

Participant to initial boxes

1. I confirm that I have read and understood the information sheet dated 22-06-18, Version 2.1 for the above study. I have had the opportunity to consider the information, ask questions and I am satisfied with the answers. ☐
2. I understand that my participation is voluntary and that I am free to withdraw from the study at any time without giving a reason. ☐
3. I understand that the information collected about me will be used to support other research in the future, and may be shared anonymously with other researchers. ☐
4. I understand that if I or someone else is believed to be in danger or at risk of significant harm, this will be reported directly to [name], [Lead for safeguarding] and Professor Claudia Estcourt, Chief Investigator on the study. Any allegations of poor practice discovered during the study will be reported directly to [name], Head of Service for GU/HIV medicine and [name], Service Manager. ☐
5. I give permission for the interview discussion to be audio-recorded and transcribed ☐
6. I agree to the use of direct quotations from the interview in publications, reports and/or presentations provided that anonymity is preserved. I understand that the results of this study may be published; however my anonymity will be preserved. ☐
7. I agree to take part in the above study. ☐
8. I confirm that I am still happy for my data to be included in the study **(to be completed at the end of the session).** ☐

________________	________________	________________
Name of participant	Date	Signature

Central and North West London NHS
NHS Foundation Trust

LUSTRUM
Limiting Undetected Sexually Transmitted Infections to RedUce Morbidity

For peer review only

[INSERT SITE LOGO]

Interview consent form (Healthcare Professionals)
Process Evaluation Study 3
Version 2.1 [22-06-18]
IRAS 000245

Name of person taking consent	Date	Signature

When completed: 1 for participant; 1 for researcher site file.

For peer review only

Central and North West London NHS
NHS Foundation Trust

LUSTRUM
Limiting Undetected Sexually Transmitted Infections to RedUce Morbidity

[INSERT SITE LOGO]

Focus group consent form (Healthcare Professionals)
Process Evaluation Study 4
Version 2.1 [22-06-18]
IRAS 000245

Appendix E

PE Study 4

Title of the project: Accelerated Partner Therapy (APT) Process Evaluation Study

Centre Number:

Study Number:

CONSENT FORM

(Healthcare Professionals –Focus Groups)

Name of Chief Investigator: Prof. Claudia Estcourt

Participant to initial boxes

1. I confirm that I have read and understood the information sheet dated 22-06-18, version 2.1 for the above study. I have had the opportunity to consider the information, ask questions and I am satisfied with the answers. ☐
2. I understand that my participation is voluntary and that I am free to withdraw from the study at any time without giving a reason. ☐
3. I understand that the information collected about me will be used to support other research in the future, and may be shared anonymously with other researchers. ☐
4. I understand that if I or someone else is believed to be in danger or at risk of significant harm, this will be reported directly to [name], [Lead for safeguarding] and Professor Claudia Estcourt, Chief Investigator on the study. Any allegations of poor practice discovered during the study will be reported directly to [name], Head of Service for GU/HIV medicine and [name], Service Manager. ☐
5. I give permission for the focus group discussion to be audio-recorded and transcribed ☐
6. I agree to the use of direct quotations from the focus group in publications, reports and/or presentations provided that anonymity is preserved. I understand that the results of this study may be published; however my anonymity will be preserved. ☐
7. I agree to take part in the above study. ☐
8. I confirm that I am still happy for my data to be included in the study (**to be completed at the end of the session**). ☐

Name of participant	Date	Signature

Central and North West London NHS
NHS Foundation Trust

LUSTRUM
Limiting Undetected Sexually Transmitted Infections to RedUce Morbidity

For peer review only

[INSERT SITE LOGO]

Focus group consent form (Healthcare Professionals)
Process Evaluation Study 4
Version 2.1 [22-06-18]
IRAS 000245

Name of person taking consent	Date	Signature

When completed: 1 for participant; 1 for researcher site file.

For peer review only

For peer review only - http://bmjopen.bmj.com/site/about/guidelines.xhtml

STANDARD PROTOCOL ITEMS: RECOMMENDATIONS FOR INTERVENTIONAL TRIALS

SPIRIT 2013 Checklist: Recommended items to address in a clinical trial protocol and related documents*

Section/item	Item No	Description	Addressed on page number
**Administrative information**			
Title	1	Descriptive title identifying the study design, population, interventions, and, if applicable, trial acronym	p1
Trial registration	2a	Trial identifier and registry name. If not yet registered, name of intended registry	p2
	2b	All items from the World Health Organization Trial Registration Data Set	Trial protocol v5.0 29.07.19
Protocol version	3	Date and version identifier	p17
Funding	4	Sources and types of financial, material, and other support	p18
Roles and responsibilities	5a	Names, affiliations, and roles of protocol contributors	p17-18
	5b	Name and contact information for the trial sponsor	p18
	5c	Role of study sponsor and funders, if any, in study design; collection, management, analysis, and interpretation of data; writing of the report; and the decision to submit the report for publication, including whether they will have ultimate authority over any of these activities	p18
	5d	Composition, roles, and responsibilities of the coordinating centre, steering committee, endpoint adjudication committee, data management team, and other individuals or groups overseeing the trial, if applicable (see Item 21a for data monitoring committee)	p13

For peer review only

Introduction

Background and rationale	6a	Description of research question and justification for undertaking the trial, including summary of relevant studies (published and unpublished) examining benefits and harms for each intervention	p3-5
	6b	Explanation for choice of comparators	p4
Objectives	7	Specific objectives or hypotheses	p5
Trial design	8	Description of trial design including type of trial (eg, parallel group, crossover, factorial, single group), allocation ratio, and framework (eg, superiority, equivalence, noninferiority, exploratory)	p5

Methods: Participants, interventions, and outcomes

Study setting	9	Description of study settings (eg, community clinic, academic hospital) and list of countries where data will be collected. Reference to where list of study sites can be obtained	p5
Eligibility criteria	10	Inclusion and exclusion criteria for participants. If applicable, eligibility criteria for study centres and individuals who will perform the interventions (eg, surgeons, psychotherapists)	p6
Interventions	11a	Interventions for each group with sufficient detail to allow replication, including how and when they will be administered	p6
	11b	Criteria for discontinuing or modifying allocated interventions for a given trial participant (eg, drug dose change in response to harms, participant request, or improving/worsening disease)	p7
	11c	Strategies to improve adherence to intervention protocols, and any procedures for monitoring adherence (eg, drug tablet return, laboratory tests)	p7
	11d	Relevant concomitant care and interventions that are permitted or prohibited during the trial	p6-7
Outcomes	12	Primary, secondary, and other outcomes, including the specific measurement variable (eg, systolic blood pressure), analysis metric (eg, change from baseline, final value, time to event), method of aggregation (eg, median, proportion), and time point for each outcome. Explanation of the clinical relevance of chosen efficacy and harm outcomes is strongly recommended	p8
Participant timeline	13	Time schedule of enrolment, interventions (including any run-ins and washouts), assessments, and visits for participants. A schematic diagram is highly recommended (see Figure)	p7; p11

Sample size	14	Estimated number of participants needed to achieve study objectives and how it was determined, including clinical and statistical assumptions supporting any sample size calculations	p8-9
Recruitment	15	Strategies for achieving adequate participant enrolment to reach target sample size	Not included

Methods: Assignment of interventions (for controlled trials)

Allocation:

Sequence generation	16a	Method of generating the allocation sequence (eg, computer-generated random numbers), and list of any factors for stratification. To reduce predictability of a random sequence, details of any planned restriction (eg, blocking) should be provided in a separate document that is unavailable to those who enrol participants or assign interventions	p9-10
Allocation concealment mechanism	16b	Mechanism of implementing the allocation sequence (eg, central telephone; sequentially numbered, opaque, sealed envelopes), describing any steps to conceal the sequence until interventions are assigned	p9-10
Implementation	16c	Who will generate the allocation sequence, who will enrol participants, and who will assign participants to interventions	p9-10
Blinding (masking)	17a	Who will be blinded after assignment to interventions (eg, trial participants, care providers, outcome assessors, data analysts), and how	p10
	17b	If blinded, circumstances under which unblinding is permissible, and procedure for revealing a participant's allocated intervention during the trial	N/A

Methods: Data collection, management, and analysis

Data collection methods	18a	Plans for assessment and collection of outcome, baseline, and other trial data, including any related processes to promote data quality (eg, duplicate measurements, training of assessors) and a description of study instruments (eg, questionnaires, laboratory tests) along with their reliability and validity, if known. Reference to where data collection forms can be found, if not in the protocol	p10
	18b	Plans to promote participant retention and complete follow-up, including list of any outcome data to be collected for participants who discontinue or deviate from intervention protocols	p7; p10-11

For peer review only - http://bmjopen.bmj.com/site/about/guidelines.xhtml

Data management	19	Plans for data entry, coding, security, and storage, including any related processes to promote data quality (eg, double data entry; range checks for data values). Reference to where details of data management procedures can be found, if not in the protocol	p10
Statistical methods	20a	Statistical methods for analysing primary and secondary outcomes. Reference to where other details of the statistical analysis plan can be found, if not in the protocol	p10-11
	20b	Methods for any additional analyses (eg, subgroup and adjusted analyses)	p11-12
	20c	Definition of analysis population relating to protocol non-adherence (eg, as randomised analysis), and any statistical methods to handle missing data (eg, multiple imputation)	p10-11
**Methods: Monitoring**			
Data monitoring	21a	Composition of data monitoring committee (DMC); summary of its role and reporting structure; statement of whether it is independent from the sponsor and competing interests; and reference to where further details about its charter can be found, if not in the protocol. Alternatively, an explanation of why a DMC is not needed	p13
	21b	Description of any interim analyses and stopping guidelines, including who will have access to these interim results and make the final decision to terminate the trial	p13
Harms	22	Plans for collecting, assessing, reporting, and managing solicited and spontaneously reported adverse events and other unintended effects of trial interventions or trial conduct	p13
Auditing	23	Frequency and procedures for auditing trial conduct, if any, and whether the process will be independent from investigators and the sponsor	Not included
**Ethics and dissemination**			
Research ethics approval	24	Plans for seeking research ethics committee/institutional review board (REC/IRB) approval	p2; p18
Protocol amendments	25	Plans for communicating important protocol modifications (eg, changes to eligibility criteria, outcomes, analyses) to relevant parties (eg, investigators, REC/IRBs, trial participants, trial registries, journals, regulators)	p13

For peer review only - http://bmjopen.bmj.com/site/about/guidelines.xhtml

Consent or assent	26a	Who will obtain informed consent or assent from potential trial participants or authorised surrogates, and how (see Item 32)	N/A
	26b	Additional consent provisions for collection and use of participant data and biological specimens in ancillary studies, if applicable	p12
Confidentiality	27	How personal information about potential and enrolled participants will be collected, shared, and maintained in order to protect confidentiality before, during, and after the trial	Not included
Declaration of interests	28	Financial and other competing interests for principal investigators for the overall trial and each study site	p18
Access to data	29	Statement of who will have access to the final trial dataset, and disclosure of contractual agreements that limit such access for investigators	p10
Ancillary and post-trial care	30	Provisions, if any, for ancillary and post-trial care, and for compensation to those who suffer harm from trial participation	N/A
Dissemination policy	31a	Plans for investigators and sponsor to communicate trial results to participants, healthcare professionals, the public, and other relevant groups (eg, via publication, reporting in results databases, or other data sharing arrangements), including any publication restrictions	p13-14
	31b	Authorship eligibility guidelines and any intended use of professional writers	p17-18
	31c	Plans, if any, for granting public access to the full protocol, participant-level dataset, and statistical code	p18
**Appendices**			
Informed consent materials	32	Model consent form and other related documentation given to participants and authorised surrogates	Trial protocol v5.0 29.07.19
Biological specimens	33	Plans for collection, laboratory evaluation, and storage of biological specimens for genetic or molecular analysis in the current trial and for future use in ancillary studies, if applicable	N/A

*It is strongly recommended that this checklist be read in conjunction with the SPIRIT 2013 Explanation & Elaboration for important clarification on the items. Amendments to the protocol should be tracked and dated. The SPIRIT checklist is copyrighted by the SPIRIT Group under the Creative Commons "Attribution-NonCommercial-NoDerivs 3.0 Unported" license.

Explainer paper - https://www.bmj.com/content/346/bmj.e7586.full?ijkey=QpAJnYI57zIwVr3&keytype=ref

For peer review only - http://bmjopen.bmj.com/site/about/guidelines.xhtml
